# The *in situ* study of surface species and structures of oxide-derived copper catalysts for electrochemical CO_2_ reduction†

**DOI:** 10.1039/d1sc00042j

**Published:** 2021-03-16

**Authors:** Chunjun Chen, Xupeng Yan, Yahui Wu, Shoujie Liu, Xiaofu Sun, Qinggong Zhu, Rongjuan Feng, Tianbin Wu, Qingli Qian, Huizhen Liu, Lirong Zheng, Jing Zhang, Buxing Han

**Affiliations:** Beijing National Laboratory for Molecular Sciences, CAS Key Laboratory of Colloid and Interface and Thermodynamics, CAS Research/Education Center for Excellence in Molecular Sciences, Institute of Chemistry, Chinese Academy of Sciences Beijing 100190 P. R. China hanbx@iccas.ac.cn; University of Chinese Academy of Sciences Beijing 100049 China; Chemistry and Chemical Engineering of Guangdong Laboratory Shantou 515063 China; Physical Science Laboratory, Huairou National Comprehensive Science Center Beijing 101400 China; Shanghai Key Laboratory of Green Chemistry and Chemical Processes, School of Chemistry and Molecular Engineering, East China Normal University Shanghai 200062 China; Institute of High Energy Physics, Chinese Academy of Sciences Beijing 100049 China

## Abstract

Oxide-derived copper (OD-Cu) has been discovered to be an effective catalyst for the electroreduction of CO_2_ to C2+ products. The structure of OD-Cu and its surface species during the reaction process are interesting topics, which have not yet been clearly discussed. Herein, *in situ* surface-enhanced Raman spectroscopy (SERS), operando X-ray absorption spectroscopy (XAS), and ^18^O isotope labeling experiments were employed to investigate the surface species and structures of OD-Cu catalysts during CO_2_ electroreduction. It was found that the OD-Cu catalysts were reduced to metallic Cu(0) in the reaction. CuO_*x*_ species existed on the catalyst surfaces during the CO_2_RR, which resulted from the adsorption of preliminary intermediates (such as *CO_2_ and *OCO^−^) on Cu instead of on the active sites of the catalyst. It was also found that abundant interfaces can be produced on OD-Cu, which can provide heterogeneous CO adsorption sites (strong binding sites and weak binding sites), leading to outstanding performance for obtaining C2+ products. The Faradaic efficiency (FE) for C2+ products reached as high as 83.8% with a current density of 341.5 mA cm^−2^ at −0.9 V *vs.* RHE.

## Abstract 2

CuO_*x*_ species were shown to exist on OD-Cu during the CO_2_RR, which resulted from the adsorption of preliminary intermediates (such as *CO_2_ and *OCO^−^) on Cu instead of on the active sites of the catalyst.

## Graphical abstract

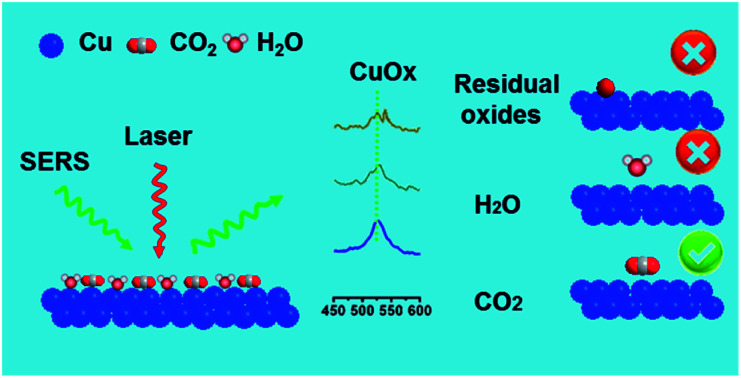

## Introduction

The electrochemical CO_2_ reduction reaction (CO_2_RR) has received wide attention, as it can not only reduce CO_2_ into chemical fuels and feedstocks but it can also provide an energy storage solution for renewable energy sources.^1–5^ In particular, multi-carbon (C2+) products are much more attractive due to their high energy densities and high economic values.^6–10^ However, the activity and selectivity for C2+ products can be severely limited due to the slow kinetics of the C–C coupling step, which involves intricate multiple proton and electron transfer.^11–15^ Designing highly active catalysts for C2+ products and confirming the active sites are crucial for promoting the development of this area.

Cu-based catalysts are the most promising electrocatalysts for converting CO_2_ to C2+ products,^16–20^ especially oxide-derived Cu (OD-Cu).^21–23^ Studies using ambient-pressure X-ray photoelectron spectroscopy, electron energy-loss spectroscopy, and *ex situ* energy-dispersive X-ray spectroscopy have shown that oxide species in OD-Cu play a crucial role in activating CO_2_ and in C–C coupling.^24–26^ However, it is well-known that Cu oxide and hydroxide species are unstable at negative potentials during the CO_2_RR. In addition, most of the characterization studies of CuO_*x*_ species are based on *ex situ* methods, and it is difficult to study the structures and valences of catalysts under reaction conditions because reduced Cu can be oxidized very rapidly, even under an atmosphere with trace amounts of O_2_. Recently, studies using *in situ* X-ray characterization have shown that the surface oxide layer can be fully reduced to metallic Cu.^27,28^ Thus, the real role of the CuO_*x*_ species of OD-Cu in promoting the CO_2_RR is controversial. In previous reports,^29,30^ massive efforts have been focused on the presence or absence of Cu oxides; however, how Cu oxides are retained or formed during the CO_2_RR is not clear. Also, comprehensive knowledge of the CuO_*x*_ species is crucial for understanding their role in promoting the CO_2_RR.

Moreover, the surface structure of a catalyst often plays a crucial role in the production of C2+ products.^31–33^ Experimental studies suggested that grain boundaries, low coordination environments, and active crystal facets can alter the CO adsorption and C–C coupling steps during the CO_2_RR.^34–37^ These factors are often associated rather than independent. For example, the active facets tend to be exposed on the surface in regions near grain boundaries, as the grain boundaries could stabilize facets with high surface energies, according to solid-state mechanical studies.^38,39^ Thus, the surface structure should also be comprehensively studied under the reaction conditions.

In this work, the surface species of OD-Cu catalysts were systematically studied *via in situ* surface-enhanced Raman spectroscopy (SERS), operando X-ray absorption spectroscopy (XAS), and isotope labeling experiments. It was found that CuO_*x*_ species existed on Cu surfaces during the CO_2_RR, and they arose from the adsorption of preliminary intermediates (such as *CO_2_ or *OCO^−^) on metallic Cu(0) at negative potentials rather than residual oxides. The presence of CuO_*x*_ species was unlikely to be the factor for enhancing C2+ product formation. Detailed experimental studies indicate that the high density of interfaces between facets played a key role in the highly efficient C2+ product production.

## Results and discussion

**Fig. 1:**
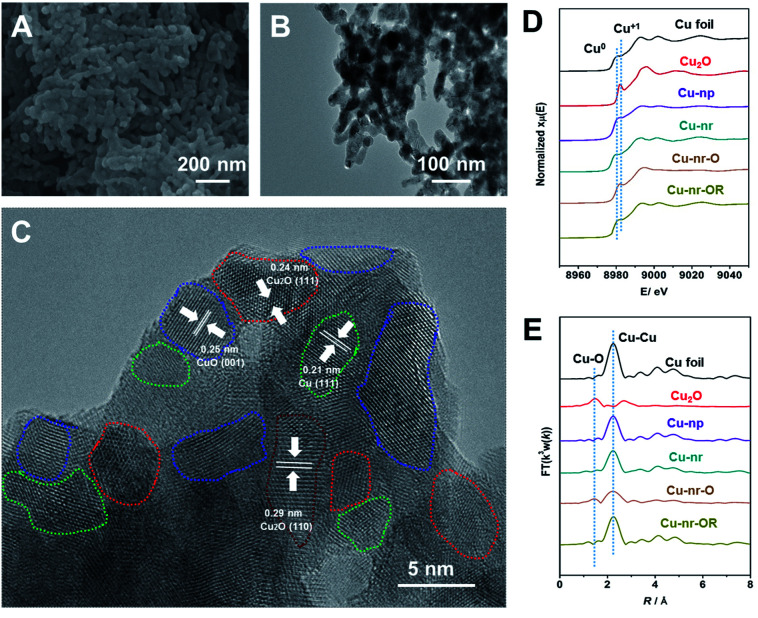
Characterization of the Cu-nr-OR catalyst. (A) An SEM image of Cu-nr-OR. (B and C) TEM and HR-TEM images of Cu-nr-OR. The areas delineated by blue, red, green, and brown dashed lines denote the CuO (100), Cu_2_O (111), Cu (111), and Cu_2_O (110) facets, respectively. (D) XANES spectra at the Cu K-edge for different catalysts. (E) The corresponding Fourier transform (FT(*k*^3^*w*(*k*))) EXAFS spectra.

Two different OD-Cu catalysts with similar morphologies were prepared in this work. Firstly, an oxide-derived Cu nanorod sample (Cu-nr) was prepared *via* the electroreduction of CuO nanorods. Another OD-Cu catalyst was prepared *via* the simple redox cycling treatment of Cu-nr. Cu-nr oxide (Cu-nr-O) was prepared *via* the oxidation of Cu-nr in 1.0 M KOH solution, then, reduced Cu-nr-O (Cu-nr-OR) was obtained *via* the electroreduction of Cu-nr-O (Fig. S1†). The obtained Cu-nr had a diameter of about 40 nm (Fig. S2†), and exhibited a cross-linked architecture. Scanning electron microscopy (SEM) and transmission electron microscopy (TEM) studies suggested that Cu-nr-OR also exhibited nanorod morphology, which was similar to Cu-nr (Fig. 1A and B). The diameters of Cu-nr and Cu-nr-OR were about 40 nm. From high-resolution TEM images (Fig. 1C and S3†), the corresponding lattice spacings of CuO (001), Cu_2_O (111), Cu_2_O (110), and Cu (111) were observed for Cu-nr-OR, which may be because Cu can be oxidized by air before measurements. In addition, a small amount of Cu_2_O can also be observed based on powder X-ray diffraction (XRD) and X-ray photoelectron spectroscopy (XPS) analysis (Fig. S4†). Furthermore, we can find that Cu-nr-OR comprised a lot of nanocrystals, and abundant interfaces between the nanocrystals were produced. Similar interfaces were found in Cu-nr (Fig. S5†), albeit the density of interfaces is significantly lower than that in Cu-nr-OR. This may be due to the size of the nanocrystals in Cu-nr being about twice that in Cu-nr-OR. Also, Cu nanoparticles (Cu-np) with a diameter of 50 nm were selected as a contrasting material. We can observe that Cu-np mostly exhibited single facets with few interfaces (Fig. S6†). These results indicated that OD-Cu exhibited abundant interfaces, which was consistent with previous reports.^21–23^

In order to obtain detailed information regarding Cu speciation, X-ray absorption spectroscopy (XAS) was used to explore the electronic structures of the catalysts. The X-ray absorption near-edge structure (XANES) spectra (Fig. 1D) showed that the pre-edge peak of Cu-nr-O was close to Cu_2_O. The oscillation *k*^3^*χ*(*k*) functions of Cu-nr-O (Fig. S7†) indicated that the low-*k* region was similar to Cu_2_O and the high-*k* region was similar to Cu, indicating that both Cu_2_O and Cu existed in Cu-nr-O. According to the extended X-ray absorption fine structure (EXAFS) spectra (Fig. 1E), Cu–O and Cu–Cu coordination peaks were observed in Cu-nr-O, implying that some Cu was oxidized to Cu_2_O. In contrast, for Cu-nr and Cu-nr-OR, only peaks corresponding to metallic Cu were observed, indicating that CuO and Cu-nr-O were fully reduced to metallic Cu after electroreduction.

**Fig. 2:**
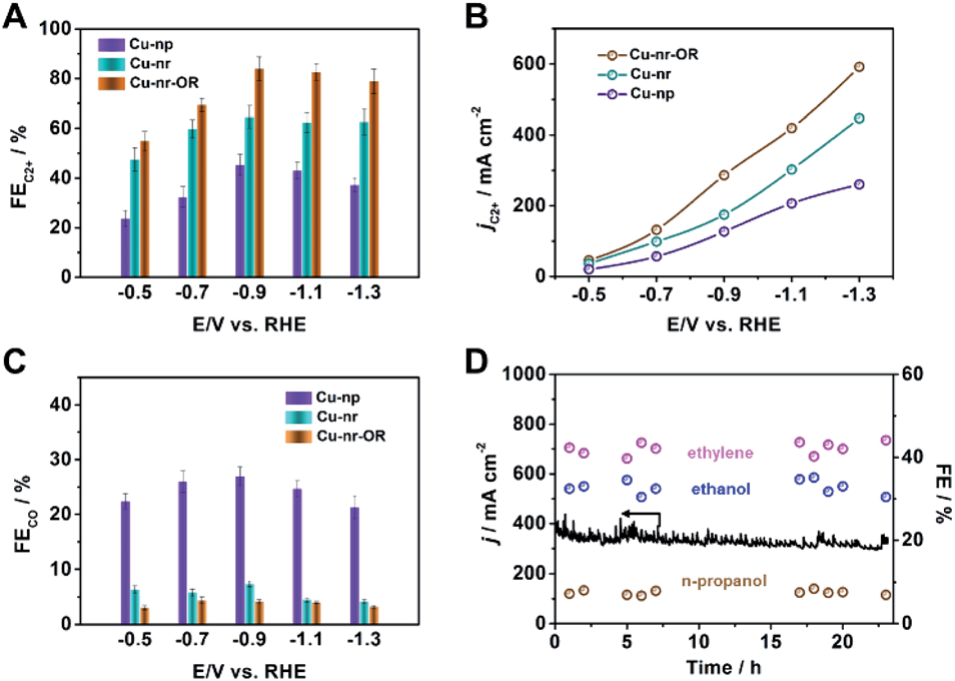
(A) The FEs for C2+ products over different catalysts in 1 M KOH solution. (B) The partial current densities for C2+ products over different catalysts in 1 M KOH solution. (C) The FEs for CO over different catalysts in 1 M KOH solution. (D) The long-term stability of Cu-nr-OR at −0.9 V *vs.* RHE for 24 h.

The electrocatalytic performances of the catalysts were evaluated in a flow cell, as reported in our previous work.^40^ Gas chromatography (GC) and nuclear magnetic resonance (NMR) spectroscopy were used to analyze the gaseous and liquid products, respectively (Fig. S8 and S9†). For Cu-nr, the FE for C2+ products (FE_C2+_) was 64.6% at −0.9 V *vs.* RHE (Fig. 2A). However, for Cu-np, FE_C2+_ was only 45.2%. It is interesting to note that Cu-nr-OR exhibited the highest FE_C2+_ value among the three catalysts, and FE_C2+_ could reach up to 83.8% with a current density of 341.5 mA cm^−2^ at −0.9 V *vs.* RHE, which was among the best values reported to date (Table S1†). The partial current density for C2+ products over Cu-nr-OR could reach 286.2 mA cm^−2^ at −0.9 V *vs.* RHE, which is about 1.6 and 2.3 times that over Cu-nr and Cu-np, respectively (Fig. 2B). Although FE_C2+_ over Cu-np was significantly lower than that over Cu-nr-OR, the FE for H_2_ was slightly higher than that over Cu-nr-OR (Fig. S10†), indicating that the increase in C2+ products for OD-Cu did not mainly arise from the suppression of the hydrogen evolution reaction (HER). We can also observe that the FE for CO over Cu-np was significantly higher than those over Cu-nr and Cu-nr-OR (Fig. 2C), indicating that generated CO can easily desorb from Cu-np, and C–C coupling can be hindered. Thus, we can assume that the low selectivity for C2+ products over Cu-np was mainly due to weak CO adsorption. The distributions of C2+ products are listed in the ESI (Fig. S11–S13†). In addition, the sums of the FEs for the different catalysts were close to 100% (Fig. S14†). Long-term operation was conducted at −0.9 V *vs.* RHE over Cu-nr-OR to elucidate the electrode stability, and the electrolyte was refreshed every hour to overcome the issue of salt accumulation.^9^ There was no obvious decay in either the current density or the FEs of the products during 24 h (Fig. 2D).

In order to verify that the products were derived from CO_2_, electrolysis experiments were conducted using isotope-labeled ^13^CO_2_ over Cu-nr-OR. From ^1^H NMR spectra (Fig. S15†), we can see that the H signals of the products split into two groups of peaks, resulting from H–^13^C coupling effects.^7,41^ In addition, we can observe that the intensities of the product signals increased with reaction time. These results indicate that CO_2_ is the only source of carbon in the products.

The intrinsic reasons for the enhanced activity and selectivity during CO_2_ reduction to C2+ products over Cu-nr-OR were further investigated. The electrochemical active surface areas (ECSAs) of Cu-np, Cu-nr, and Cu-nr-OR were estimated *via* measuring the double-layer capacitance and using the Pb underpotential deposition (Pb_UPD_) method (Fig. S16 and S17†). We can observe that the ECSA of Cu-nr-OR was similar to that of Cu-nr, and it was higher than that of Cu-np. Similar to the geometric current density trend, Cu-nr-OR also exhibited the highest normalized current density among the three samples (Fig. S18 and Table S2†). Thus, the improved C2+ product generation did not mainly result from a change in the ECSA. Furthermore, electrochemical impedance spectroscopy (EIS) was carried out to measure the charge transfer resistance (*R*_ct_) values of Cu-nr and Cu-nr-OR. Cu-nr-OR showed a similar interfacial *R*_ct_ value as Cu-np and Cu-nr (Fig. S19†).

**Fig. 3:**
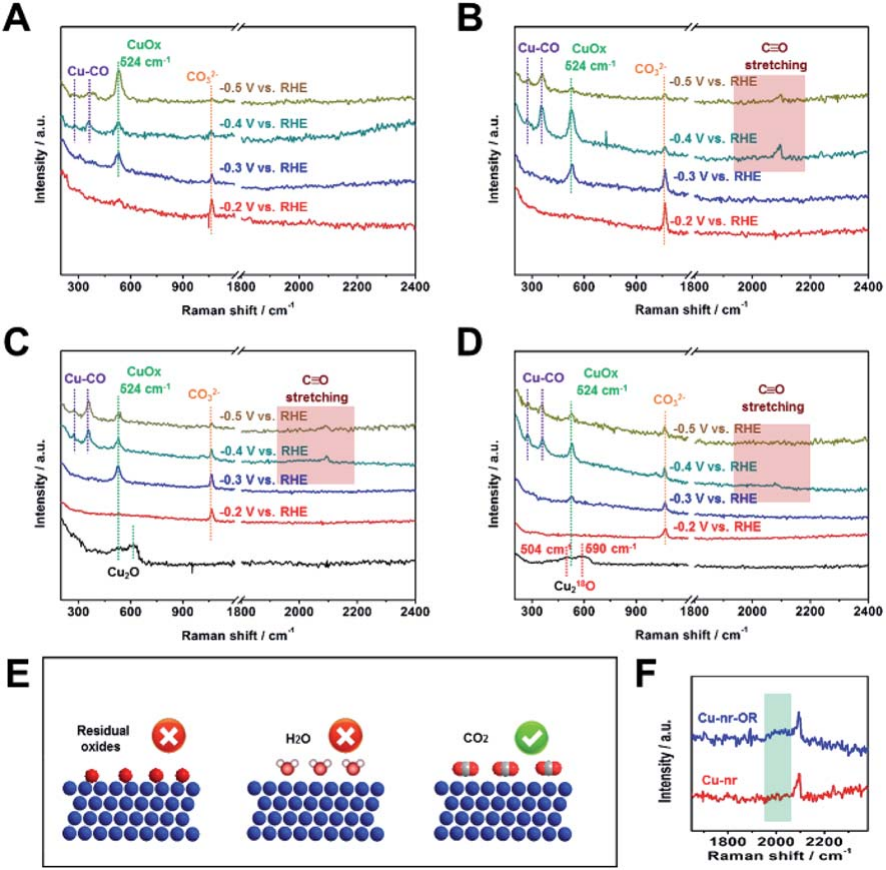
The *in situ* surface-enhanced Raman spectra of Cu-np (A), Cu-nr (B), and Cu-nr-OR (C) at different potentials during the CO_2_RR. (D) The *in situ* surface-enhanced Raman spectra of ^18^O-enriched Cu-nr-O at different potentials during the CO_2_RR. (E) A schematic illustration of the possible reasons for the formation of CuO_*x*_ species during the CO_2_RR. (F) A local enlarged view of the *in situ* surface-enhanced Raman spectra of Cu-nr and Cu-nr-OR at −0.4 V *vs.* RHE during the CO_2_RR.

Due to the C2+ product selectivity and activity being sensitive to the surface species of catalysts, the role of surface species in enhancing the generation of C2+ products was explored *via in situ* SERS (Fig. S20†). For Cu-np, Cu-nr, and Cu-nr-OR, no bands corresponding to Cu oxides (CuO or Cu_2_O) can be observed at −0.2 V *vs.* RHE or below, indicating the full reduction of Cu oxides to metallic Cu (Fig. 3A–C); this was consistent with the results of XAFS. In addition, from cyclic voltammetry curves (Fig. S21†), no oxide peaks can be observed at low potentials. New bands appeared at −0.2 V *vs.* RHE or below during the CO_2_RR, and the bands will be analyzed according to the different potentials below.

At −0.2 V *vs.* RHE or below, well-defined bands appeared at 1064 cm^−1^ for Cu-np, Cu-nr, and Cu-nr-OR during the CO_2_RR, which were attribution to CO_3_^2−^,^12^ because CO_2_ can dissolve in KOH electrolyte, forming a neutral-pH carbonate mixture. The intensity of the band became weaker as the potential decreased, which is because CO_2_ can be reduced at negative potential and the formation of CO_3_^2−^ becomes slow.

At −0.3 V *vs.* RHE or below, an additional band appeared at 524 cm^−1^ for Cu-np, Cu-nr, and Cu-nr-OR during the CO_2_RR. According to a previous report,^42^ the band can be assigned to CuO_*x*_ or CuO_*x*_OH_*y*_ species. When analysis was conducted in D_2_O instead of H_2_O, the band at 524 cm^−1^ displayed a negligible shift (Fig. S22†), indicating that the band could be attributed to CuO_*x*_ species rather than CuO_*x*_OH_*y*_.^42^ There are three possible reasons for the formation of CuO_*x*_ species (Fig. 3E): (1) the CuO_*x*_ species were from the original Cu_2_O; (2) they arose from a reaction between reduced Cu and H_2_O; and (3) they arose from a reaction between reduced Cu and CO_2_.

To answer this question, ^18^O isotope labeling experiments were carried out to confirm the source of oxygen in CuO_*x*_. First, ^18^O-enriched Cu-nr-O catalysts were synthesized *via* the oxidation of Cu-nr in H_2_^18^O electrolyte. Cu_2_^18^O was formed in Cu-nr-O; the bands were at 504 and 590 cm^−1^ (Fig. 3D), exhibiting significant red shifts compared with Cu_2_^16^O.^29^ This result indicates that the bands of CuO_*x*_ species showed significant red shifts when ^16^O was replaced with ^18^O, which can be an important indicator when exploring the oxygen source in the CuO_*x*_ species. For ^18^O-enriched Cu-nr-OR, CuO_*x*_ was also formed at −0.3 V *vs.* RHE, and the band remained at 524 cm^−1^, displaying a negligible shift compared with that of Cu-nr-OR. The results suggest that the CuO_*x*_ species were not from the original Cu_2_^18^O. Thus, it can be deduced that CuO_*x*_ was produced during the CO_2_RR. Because both H_2_O and CO_2_ can react with reduced Cu to form CuO_*x*_ species, the oxygen source of the CuO_*x*_ species should be further studied. Furthermore, Cu-nr-OR was tested in H_2_^18^O during the CO_2_RR; we observed that the band of CuO_*x*_ was still at 524 cm^−1^ (Fig. S23†), which indicated that CuO_*x*_ did not arise from a reaction between Cu and H_2_^18^O. Thus, the last possible reason is reasonable, *i.e.*, CuO_*x*_ arose from a reaction between Cu and CO_2_. To further verify this argument, electrolysis experiments using Cu-nr-OR were carried out under a N_2_ atmosphere; no bands were observed at a negative potential (Fig. S24†), indicating that CO_2_ played a key role in the formation of CuO_*x*_ species. In addition, the CuO_*x*_ band cannot be observed at −0.2 V *vs.* RHE, and it appeared at −0.3 V *vs.* RHE or below. Thus, we can assume that this peak cannot be attributed to the simple adsorption of CO_2_ on Cu. CO_2_ can be reduced to preliminary intermediates at low potentials; thus, we can assume that the CuO_*x*_ species were from the adsorption of preliminary intermediates (such as *CO_2_ and *OCO^−^) on Cu, which has been observed on Ag metal.^43^

From the above results, we can deduce that CuO_*x*_ that existed during the CO_2_RR was just a signal of the chemisorption of CO_2_ on Cu, which was not the main factor for facilitating C2+ product formation during the CO_2_RR. And the CuO_*x*_ species were not specific to the Cu-oxide derived catalysts.

At −0.4 V *vs.* RHE or below, for Cu-np, Cu-nr, and Cu-nr-OR, the presence of adsorbed *CO on Cu was demonstrated by the appearance of Raman peaks located at 276, 360, and 2000–2100 cm^−1^, which correspond to the restricted rotation of adsorbed *CO on Cu, Cu–CO stretching, and C≡O stretching, respectively.^44^ We can observe that these peaks were significantly weaker for Cu-np than for Cu-nr and Cu-nr-OR, which can result from the weak adsorption of CO or rapid reactions on Cu-np. From the results of the CO_2_RR (Fig. 2A and C), we know that the FE for CO over Cu-np was higher than those over Cu-nr and Cu-nr-OR, and the FE for C2+ products over Cu-np was lower than those over Cu-nr and Cu-nr-OR; thus, we can assume that the weak peaks on Cu-np could be attributed to the weak adsorption of CO on Cu-np. It is interesting to note that the C≡O stretching band of Cu-nr-OR is different from that of Cu-nr (Fig. 3F). A new peak appeared at about 2000 cm^−2^ in the case of Cu-nr-OR compared with Cu-nr. Specifically, the stretching mode of surface-adsorbed CO can serve as a molecular probe of the surface structure due to its sensitivity to the structures of adsorption sites.^45,46^ It is reasonable to analyze the active sites using surface-adsorbed CO at −0.4 V *vs.* RHE, because the FE for C2+ products is low (Fig. S25†), indicating that the C–C coupling step is slow at this potential. Thus, we can assume that new adsorption sites for CO were produced on Cu-nr-OR. Also, the new sites exhibited slightly weaker adsorption for CO than Cu-nr, due to the band having a lower wavenumber than that of Cu-nr. Therefore, Cu-nr-OR exhibited heterogeneous CO adsorption sites, which included stronger binding sites and weaker binding sites. According to previous reports,^47,48^ the energy barrier of C–C coupling can be decreased on stronger and weaker CO binding sites. Thus, we can suppose that the enhanced generation of C2+ products over Cu-nr-OR originated mainly from the formation of heterogeneous CO adsorption sites. Combining the HR-TEM results, we can assume that the heterogeneous CO adsorption sites could be attributed to the abundant interfaces of Cu-nr-OR.

After the potential was removed, we could observe that Cu_2_O was formed rapidly (Fig. S26†), indicating that the reduced Cu can be oxidized in electrolyte. Cu_2_O can be formed *via* the oxidation of Cu by O_2_ in the electrolyte.^29^ However, the O_2_ content is very low in the cathodic electrolyte. It is interesting to note that Cu_2_^18^O was formed when using H_2_^18^O as the electrolyte after the potential was removed (Fig. S27†). Thus, we can assume that Cu_2_O arose from the reaction between Cu and H_2_O. It is known that the oxidation of Cu by water is a thermodynamically unfavorable process. Thus, only a small proportion of Cu can be oxidized. The results indicate that the *in situ* method has obvious advantages and is necessary when exploring the real state of a catalyst during a reaction.

**Fig. 4:**
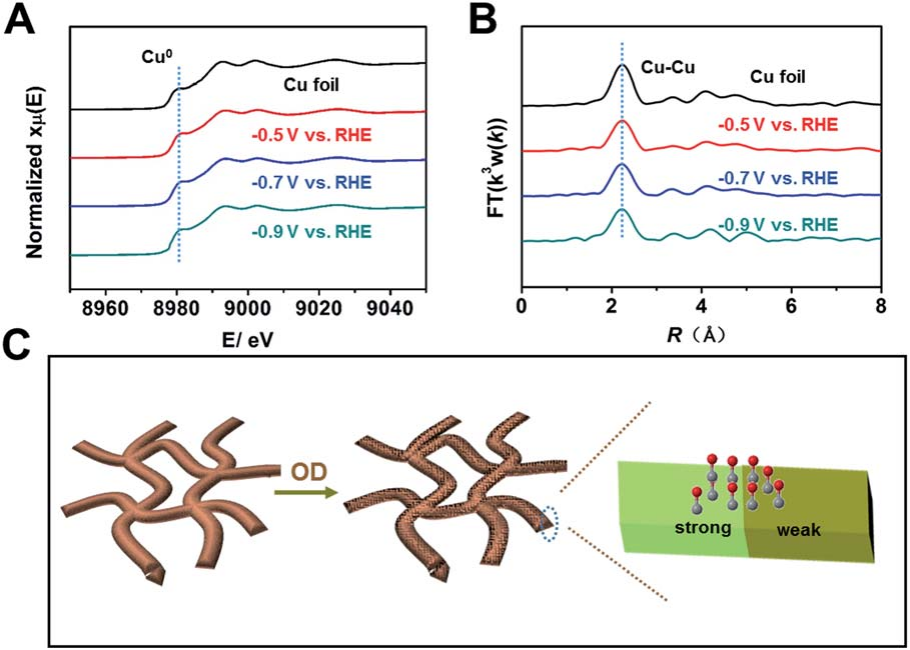
(A) Operando XANES spectra at the Cu K-edge of Cu-nr-OR at different potentials during the CO_2_RR. (B) The corresponding Fourier transform (FT(*k*^3^*w*(*k*))) EXAFS spectra of Cu-nr-OR at different potentials during the CO_2_RR. (C) A schematic illustration of the reason for the outstanding C2+ production performance over Cu-nr-OR.

Because the coordination environment of Cu can alter the adsorption of CO and the energy barrier of the C–C coupling step, we used operando XAS to monitor the local structures of Cu-np, Cu-nr, and Cu-nr-OR during the CO_2_RR (Fig. S28†). For both catalysts, only peaks corresponding to metallic Cu were observed at negative potentials during the CO_2_RR (Fig. 4A, B and S29–S31†), indicating that CuO or Cu_2_O was reduced to metallic Cu in the CO_2_RR. Moreover, the quantified Cu–Cu coordination numbers of Cu-nr-OR, Cu-nr, and Cu-np were fit using the ARTEMIS programs of IFEFFIT during the CO_2_RR (Fig. S32–S34 and Table S3†). No obvious differences in the Cu–Cu coordination numbers were observed for Cu-nr-OR, Cu-nr, and Cu-np during the CO_2_RR, indicating that the enhanced C2+ product formation over Cu-nr-OR did not mainly arise from the slight change in the coordination environment.

From the above results, we can make the conclusion that abundant interfaces can be produced on Cu-nr-OR, and stronger and weaker binding sites for CO appeared at the abundant interfaces, which can decrease the energy barrier of C–C coupling (Fig. 4C). Also, the coordination environment of Cu-nr-OR does not show obvious changes compared with Cu-nr. Thus, the increased interfaces can be considered as the main factor for the enhanced generation of C2+ products over Cu-nr-OR.

## Conclusions

In summary, the surface species and structures of OD-Cu catalysts were systematically investigated *via in situ* SERS, operando XAS, and ^18^O isotope labeling experiments. It was shown that Cu oxides indeed existed on the surfaces of the catalysts during the CO_2_RR. However, they were formed *via* the adsorption of preliminary intermediates (such as *CO_2_ and *OCO^−^) on Cu instead of on the active sites of the catalyst. Abundant interfaces can be produced on Cu-nr-OR, which can provide heterogeneous CO adsorption sites, enhancing the C2+ product selectivity. In addition, this work also shows that *in situ* techniques have obvious advantages and are sometimes necessary for exploring the structures of catalysts and the surface species during the CO_2_RR. We believe that the findings of this work provide useful knowledge for designing other efficient electrocatalysts for CO_2_ reduction.

## Author contributions

Chunjun Chen and Buxing Han proposed the project, designed the experiments and wrote the manuscript; Chunjun Chen performed the whole experiments; Xupeng Yan, Yahui Wu, Huizhen Liu, Xiaofu Sun, Qinggong Zhu, Tianbin Wu and Qingli Qian assisted in analyzing the experimental data; Fengrong Juan assisted in analyzing the experimental data of *in situ*-SERS; Shoujie Liu, Jing Zhang and Lirong Zheng assisted in analyzing the experimental data of XAS; Buxing Han supervised the whole project.

## Conflicts of interest

There are no conflicts to declare.

## Supplementary Material

SC-012-D1SC00042J-s001
