# Supplementary material for: The in situ study of surface species and structures of oxide-derived copper catalysts for electrochemical CO2 reduction
Source: Chem Sci. 2021 Mar 16;12(16):5938–43. doi: 10.1039/d1sc00042j (PMC8869928; doi:10.1039/d1sc00042j)
Supplement: SC-012-D1SC00042J-s001 [file SC-012-D1SC00042J-s001.pdf]

Electronic Supplementary Material (ESI) for Chemical Science.
This journal is © The Royal Society of Chemistry 2021

In situ study of surface species and structure over Oxide-Derived Copper Catalysts for Electrochemical CO_2 Reduction

Chunjun Chen,[a,b] Xupeng Yan,[a,b] Yahui Wu,[a,b] Shoujie Liu,[c] Xiaofu Sun,[a,b] Qinggong Zhu,[a] Rongjuan Feng,[a] Tianbin Wu, [a] Qingli Qian,[a] Huizhen Liu,[a] Lirong Zheng,[f] Jing Zhang,[f] and Buxing Han[a,b,d,e]*

a Beijing National Laboratory for Molecular Sciences, Key Laboratory of Colloid and Interface and Thermodynamics, Institute of Chemistry, Chinese Academy of Sciences, Beijing 100190, P. R. China

b School of Chemistry and Chemical Engineering, University of Chinese Academy of Sciences, Beijing 100049, P. R. China

c Chemistry and Chemical Engineering of Guangdong Laboratory, Shantou 515063, China.

d Physical Science Laboratory, Huairou National Comprehensive Science Center, No. 5 Yanqi East Second Street, Beijing 101400, P. R.China

e Shanghai Key Laboratory of Green Chemistry and Chemical Processes, School of Chemistry and Molecular Engineering, East China Normal University, Shanghai 200062, P. R. China

f Institute of High Energy Physics, Chinese Academy of Sciences, Beijing 100049, China.

Experimental Section

Materials: $Cu(NO_3)_2$ (99%), NaOH (98%), ammonium hydroxide (30%), $HClO_4$ (70-72 wt%) and Ni foam were obtained from Sinopharm Chem. Reagent Co. Ltd. KOH (98%), sodium 2, 2-dimethyl-2-silapentane-5-sulfonate (DSS, 99%), Cu nanoparticle (50nm), $Pb(ClO_4)_2$ and phenol were purchased from Alfa Aesar China Co., Ltd. D_2O (99.8% D) and $H_2{}^{18}O$ (97% ^{18}O) were purchased from Innochem Co., Ltd. N_2 (99.999%) and CO_2 (99.999%) were provided by Beijing Analytical Instrument Company. Deionized water was used in the experiments.

Synthetic procedures for Cu nanorods (Cu-nr): The Cu-nr was prepared by electroreduction of the CuO nanorods (CuO-nr) for 10 min at -0.9 V vs. RHE. CuO-nr was fabricated through the annealing of $Cu(OH)_2$ nanorods under N_2 atmosphere. Firstly, the $Cu(OH)_2$ nanorods were prepared by a literature method.[S1] Typically, 1 g of $Cu(NO_3)_2$ was dissolved in 100 mL distilled water. Then, 30 mL $NH_3 \cdot H_2O$ (0.15 M) solution was added to the $Cu(NO_3)_2$ solution under constant stirring at room temperature. A blue precipitate of $Cu(OH)_2$ was produced when 10 mL of NaOH (1 M) solution (≈2 mL min^{-1}) was dropwise added to the above solution to adjust the pH value to 9-10. After 30 min, the blue $Cu(OH)_2$ precipitate was centrifuged and washed several times with H_2O to obtain a solid product, which was dried by freeze-drying for 24 h. Secondly, the CuO-nr was prepared by annealing the $Cu(OH)_2$ nanorods in the N_2 atmosphere at 500 °C for 2 h with a heating rate of 20 °C min^{-1}. Finally, the Cu-nr was prepared by electroreduction of the CuO nanorods (CuO-nr) for 10 min at -0.9 V vs. RHE, and the electrolyte used was 1M KOH solution.

Synthetic procedures for Cu-nr-OR： The Cu-nr-OR was prepared by electroreduction of the Cu-nr-O for 10 min at -0.9 V vs. RHE. Firstly, the Cu-nr-O was prepared by electrochemical cycling of Cu-nr in 1.0 M KOH solutions. The experiment was performed in multi-potential steps mode. The potential and time for the step 1 was 1.0 V vs. RHE and 2s; the potential and time for the step 2 was 0.4 V vs. RHE and 1s; Then the Cu-nr-O was obtained after 20 cycles. Secondly, the Cu-nr-OR was prepared by electroreduction of the Cu-nr-O for 10 min at -0.9 V vs. RHE, and the electrolytewas 1M KOH solution.

Characterization of the materials. The SEM and TEM characterizations were carried out using a HITACHI S-4800 and JEOL JEM-2100F, equipped with EDS. The operando X-ray adsorption spectroscopy (XAS) measurements were performed using a modified flow cell at the 1W1B, 1W2B beamline at Beijing Synchrotron Radiation Facility (BSRF). In situ Raman measurements were carried out using a Horiba LabRAM HR Evolution Raman microscope in a modified flow cell, which was produced by GaossUnion (Tianjin) Photoelectric Technology Company (Figure S10). A 785-nm laser was used and signals were recorded using a 20 s integration and by averaging two scans.

Preparation of electrodes. To construct the cathode electrode, a catalyst slurry that contained 5 mg of obtained catalysts, 1 mL of methanol and 20 μL of Nafion ionomer solution (5 wt% in H_2O) was first mixed and sonicated for 30 min. Then, the catalyst slurry (0.2 mL) was slowly drop cast onto a PTFE membrane (Fuel Cell Store) under vacuum to achieve a catalyst loading of ~1.0 mg cm^{-2}. Ni foam was used as anode electrode.

Electrochemical study. Electrochemical studies were conducted in an electrochemical flow cell which including a gas chamber, a cathodic chamber, and an anodic chamber, as reported in our previous work.[S2] An anion exchange membrane (FumasepFAA-3-PK-130) was used to separate the anodic and cathodic chambers, and an Ag/AgCl electrode and Ni foam were used as the reference and counter electrodes, respectively. The electrolysis was conducted using a CHI 660e electrochemical workstation equipped with a high current amplifier CHI 680c. The measured potentials after iR compensation were rescaled to the RHE by E (versus RHE) = E (versus Ag/AgCl) + 0.209 V+0.0591V/pH×pH. For performance studies, 1 M KOH was used as the electrolyte, and it was circulated through the cathodic and anodic chambers using peristaltic pumps at a rate of 20 mL min^{-1}. The flow rate of CO_2 gas through the gas chamber was controlled to be 20 sccm using a digital gas flow controller.

EIS study. The EIS measurement was carried out in 1 M KOH solution at an open circuit potential (OCP) with an amplitude of 5 mV of 10^{-2} to 10^{6} Hz.

Double-layer capacitance (C_{dl}) measurements. The electrochemical active surface area is proportional to C_{dl} value. C_{dl} was determined by measuring the capacitive current associated with double-layer charging from the scan-rate dependence of cyclic voltammogram (CV). The CV ranged from 0 V to -0.1 V vs. RHE. The C_{dl} was estimated by plotting the Δj (j_a-j_c) at -0.05 V vs. RHE against the scan rates, in which the j_a and j_c are the anodic and cathodic current density, respectively. The scan rates were 20, 30, 50, 80, 100 and 120 mV s^{-1}.

Pb underpotential deposition: Cu_{ECSA} in catalysts was determined using Pb underpotential deposition. An N_2-saturated solution of 100 mM $HClO_4$ + 1 mM $Pb(ClO_4)_2$ was used as the electrolyte. The cathode was held at -0.081 VRHE for 60 s and then cyclic voltammetry was recorded between -0.5 and 0.2 VRHE at 5 mV s^{-1}. Pt foil was used as the anode. The electrolyte was not circulated during the cyclic voltammetry measurement. The Cu_{ECSA} in the catalyst is calculated from the charge associated with 2e^- oxidation of monolayer of Pb adatoms coverage over Cu surface.

Product analysis. The gaseous product of electrochemical experiments was collected using a gas bag and analyzed by gas chromatography (GC, HP 4890D), which was equipped with TCD detectors using argon as the carrier gas. The liquid product was analyzed by ^{1}H NMR (Bruker Avance III 400 HD spectrometer) in deuteroxide.

Calculations of Faradaic efficiencies of gaseous and liquid products.

liquid products:

After electrolysis, a certain amount of internal standard solution was added to the electrolyte as the internal standard. Because the concentration of internal standard was known, the moles of liquid products can be calculated from integral areas and calibration curves. To accurately integerate the products in NMR analysis, two standards located in different regions were used in NMR analysis. The sodium 2, 2-dimethyl-2-silapentane-5-sulfonate (DSS) was the reference for n-propanol, ethanol and acetic acid, and the phenol was the reference for formate. 400 μL catholyte after the reaction was mixed with 100 μL 6 mM DSS solution, 100 μL 200 mM phenol and 200 μL D_2O, and then analyzed by 1H NMR (Bruker Avance III 400 HD spectrometer).

The Faradaic efficiency of liquid product is:

$$FE = \frac{moles\ of\ product}{Q\ /\ nF} \times 100\%$$

(Q: charge (C); F：Faradaic constant (96485 C/mol); n: the number of electrons required to generate the product)

Gaseous products:

From the GC peak areas and calibration curves for the TCD detector, we can obtain the V % of gaseous products . Since the flow rate of the CO_2 was constant, the moles of gaseous products can be calculated. The Faradaic efficiency of gaseous product is:

$$FE = \frac{moles\ of\ product}{Q\ /\ nF} \times 100\%$$

(Q: charge (C); F：Faradaic constant (96485 C/mol); n: the number of electrons required to generate the product)

Supplementary Figures

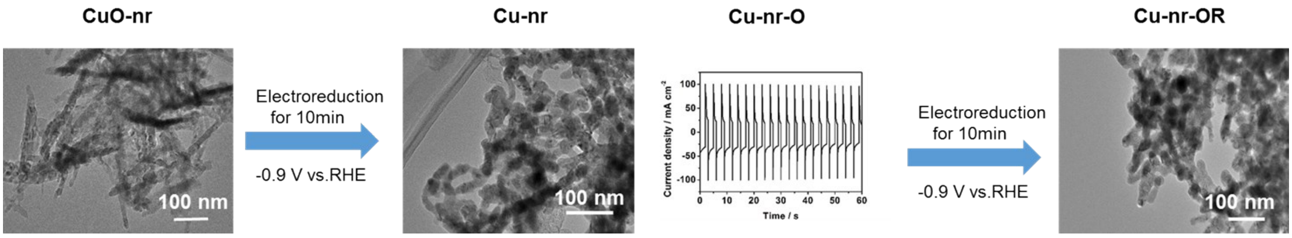

Figure S1. The schematic illustration for the formation of Cu-nr-OR.

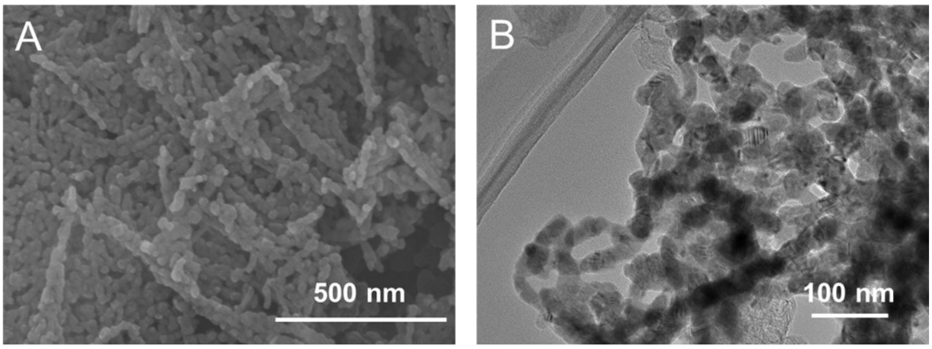

Figure S2. (A) The SEM images of the Cu-nr; (B) The TEM image of the Cu-nr.

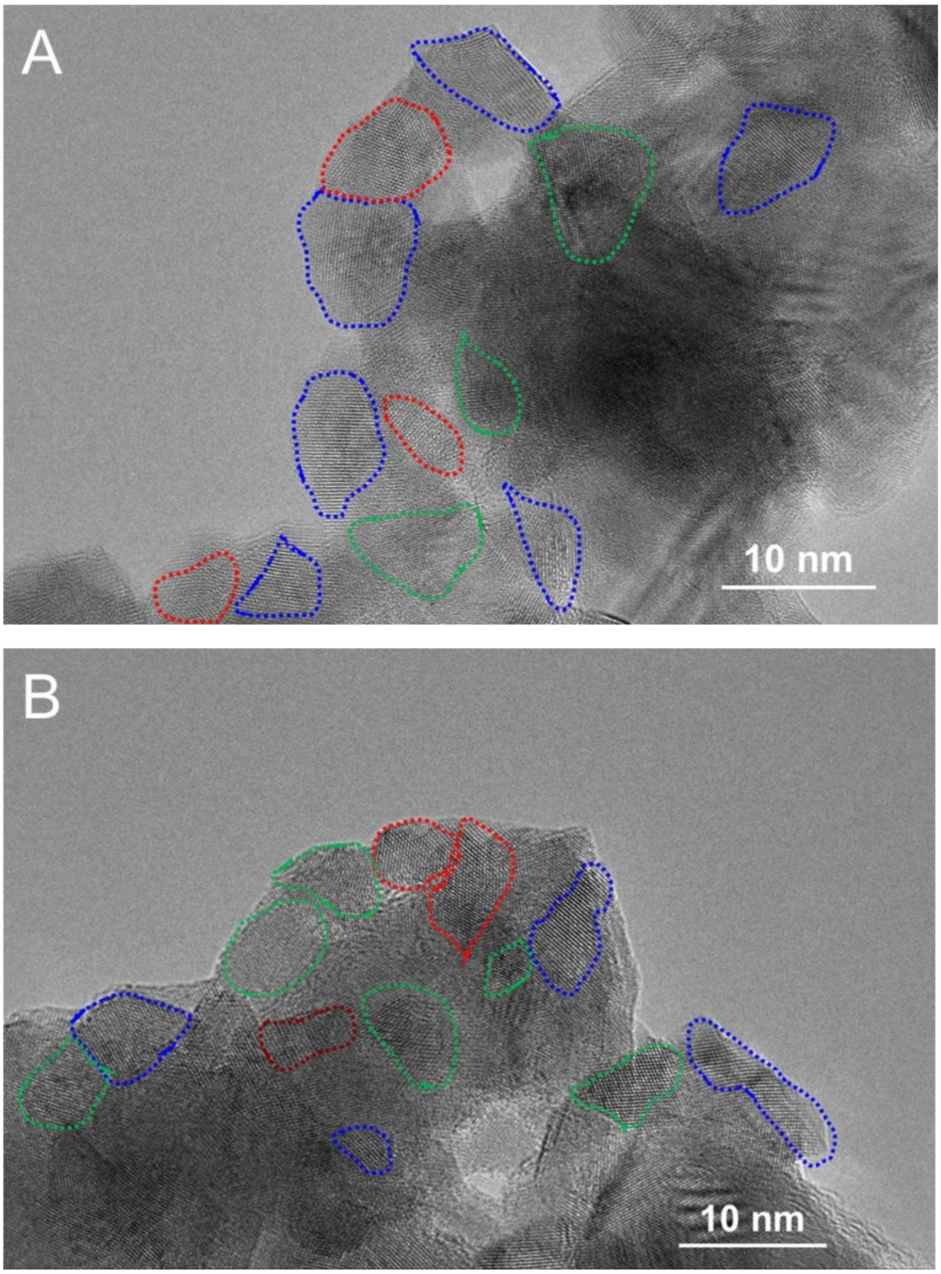

Figure S3. The HR-TEM images of the Cu-nr-OR. The areas delineated by blue, red, green and brownness dashed lines denote the CuO (100), Cu_2O (111), Cu (111) and Cu_2O (110) facets, respectively.

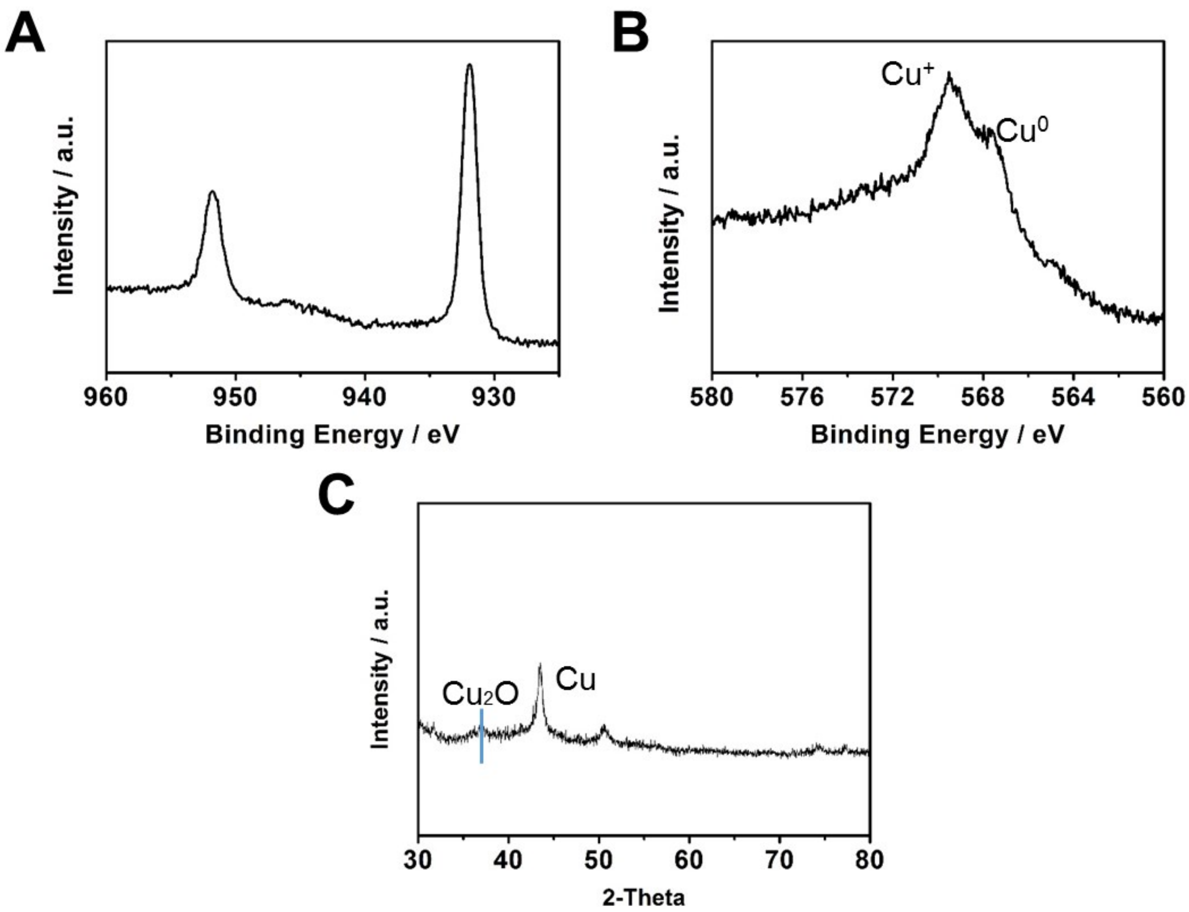

Figure S4. (A) The Cu 2p XPS spectra of Cu-nr-OR. (B) The Cu LMN spectra of Cu-nr-OR. (C) The XRD of the Cu-nr-OR.

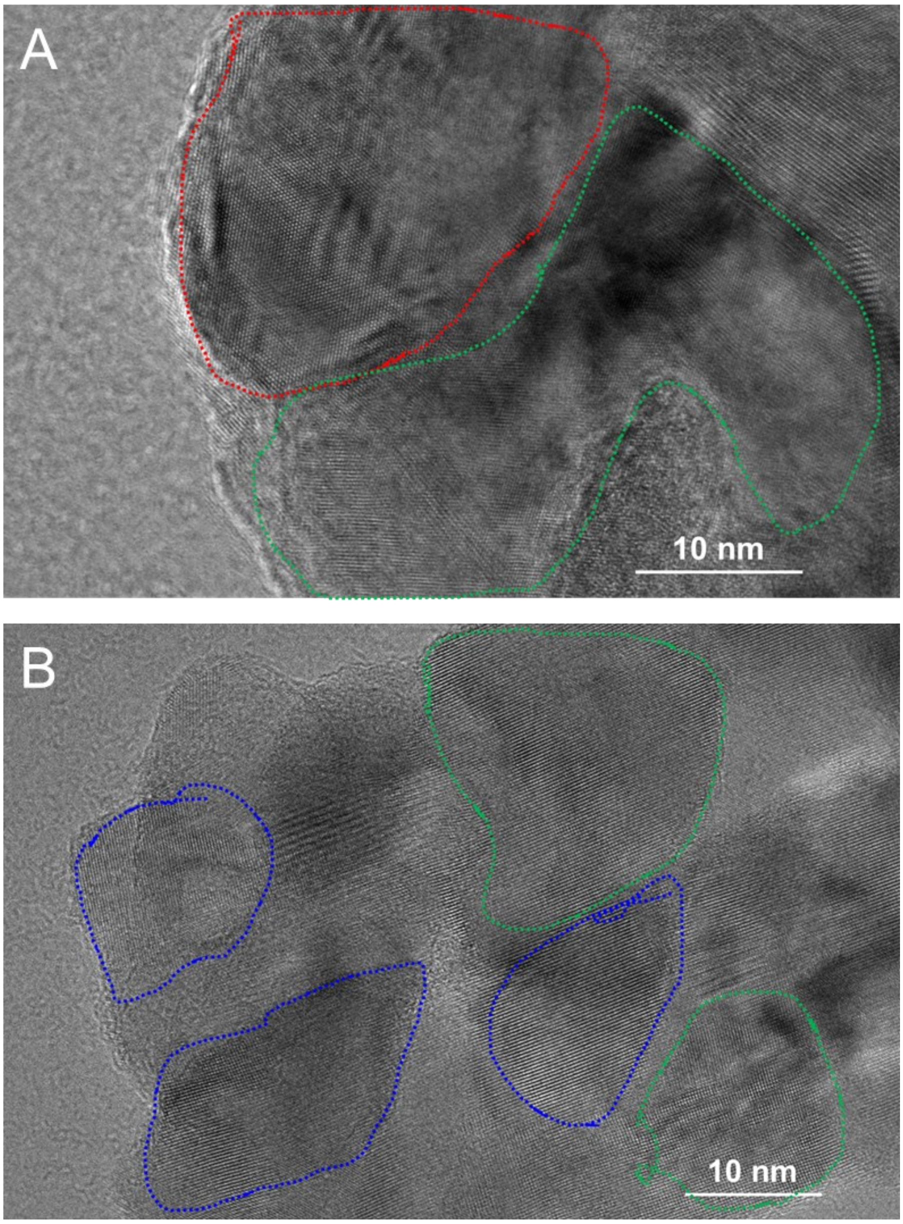

Figure S5. The HR-TEM images of the Cu-nr. The areas delineated by blue, red, green and brownness dashed lines denote the CuO (100), Cu_2O (111), Cu (111) and Cu_2O (110) facets, respectively.

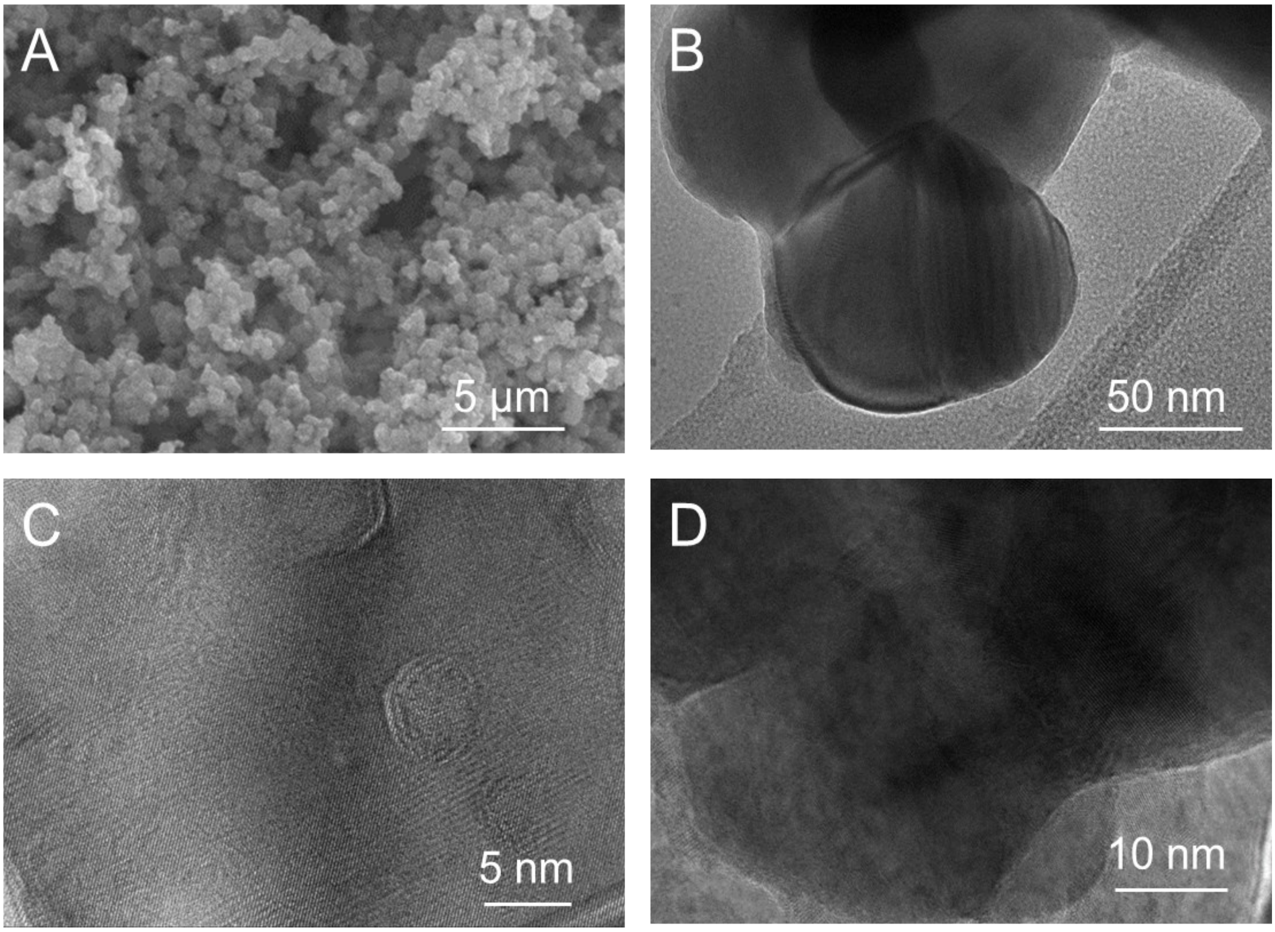

Figure S6. (A) The SEM images of the Cu-np; (B) The TEM image of the Cu-np; (C, D) The HR-TEM images of the Cu-np.

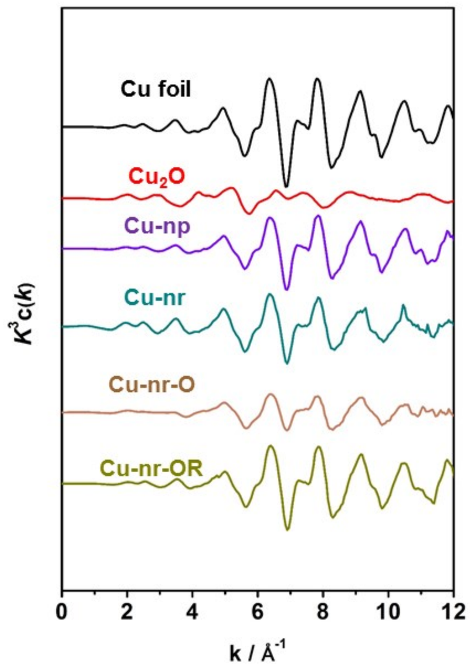

Figure S7. Cu K-edge extended XAFS oscillation function $k^3w(k)$ for various catalysts.

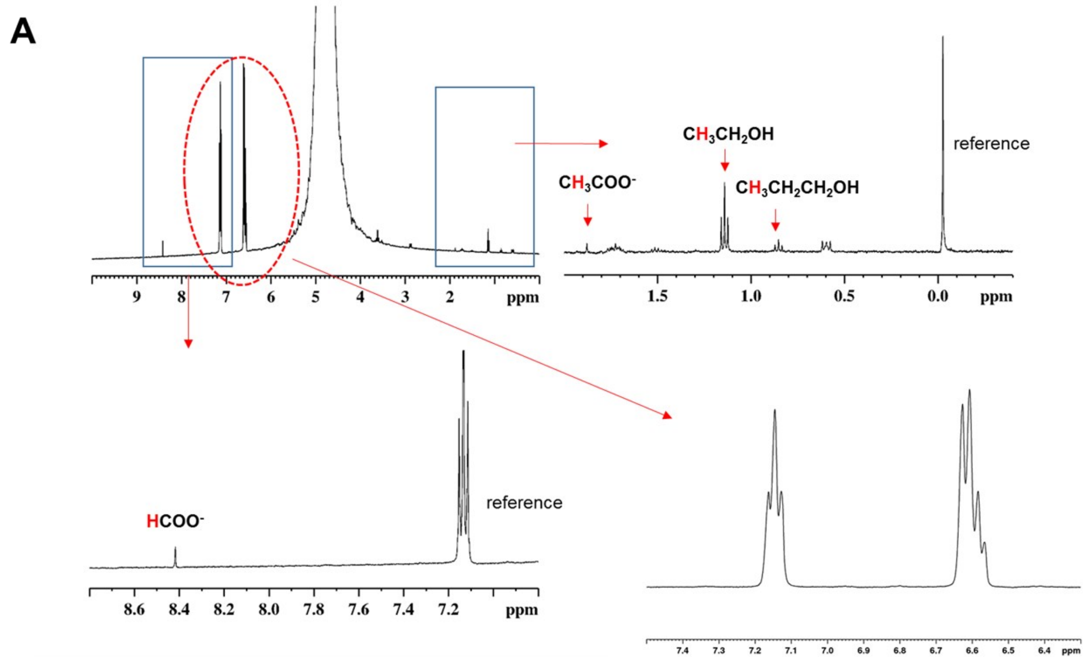

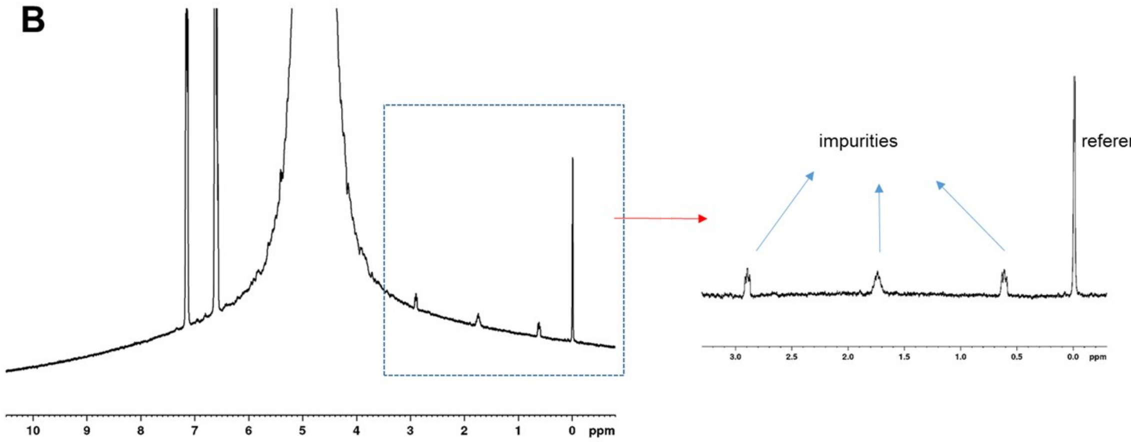

Figure S8. (A) A typical ^{1}H NMR spectrum of liquid products after electrolysis over the Cu-nr-OR; (B) ^{1}H NMR spectrum of the reference.

In our work, the DSS was used as internal standard, and there was a small amount of impurity in DSS, the signal at 0.6 ppm, 1.7 ppm and 2.9 ppm were assigned to the impurity.

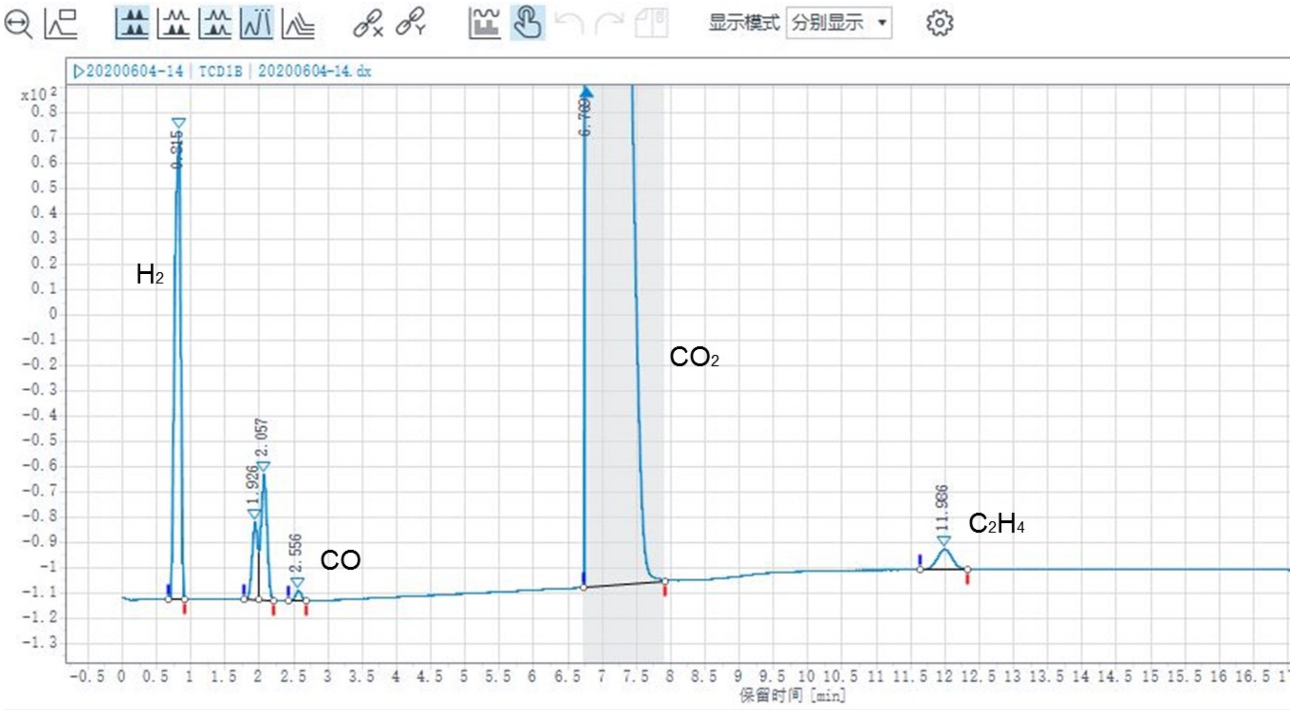

Figure S9. A typical GC spectrum of as products after electrolysis over the Cu-nr-OR.

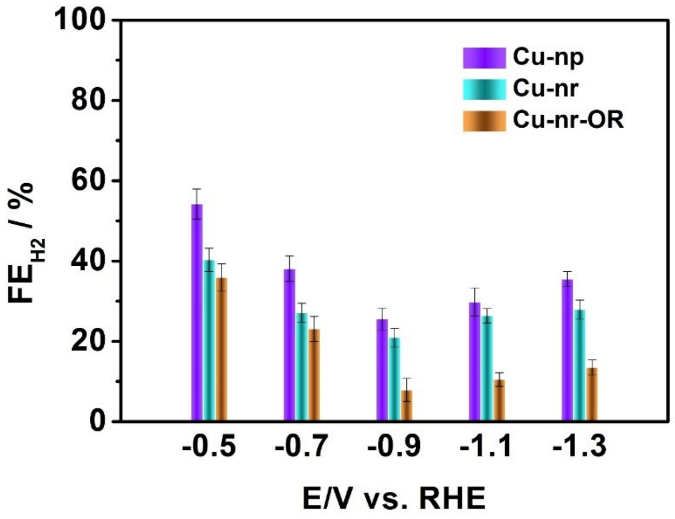

Figure S10 The FE of H_2 for Cu-np, Cu-nr and Cu-nr-OR.

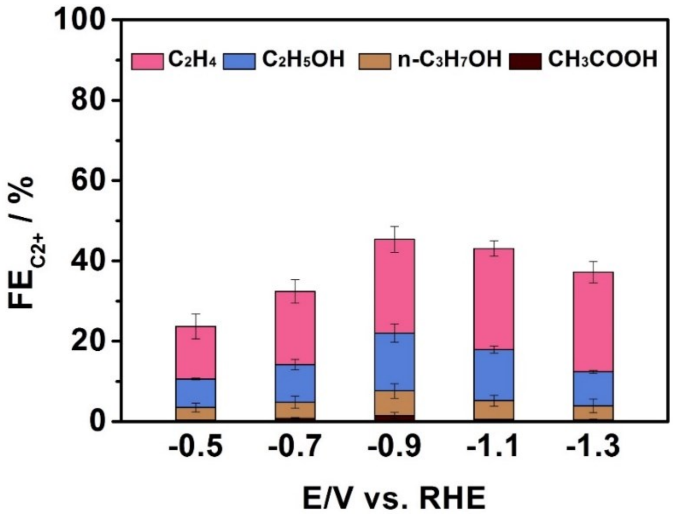

Figure S11. The distribution of C2+ products for Cu-np.

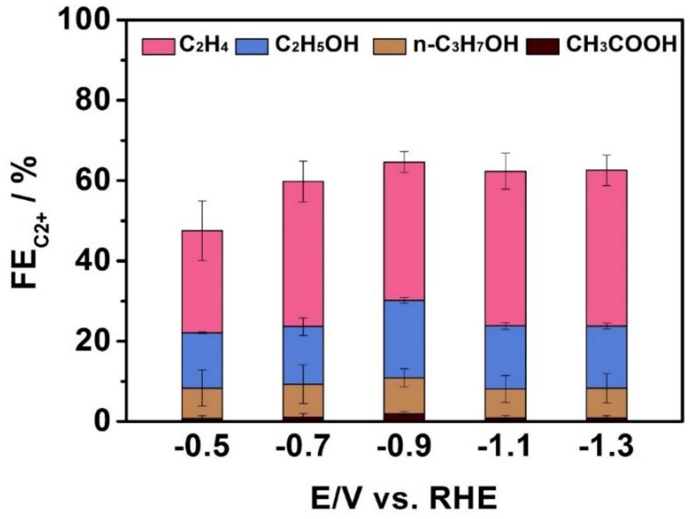

Figure S12. The distribution of C2+ products for Cu-nr.

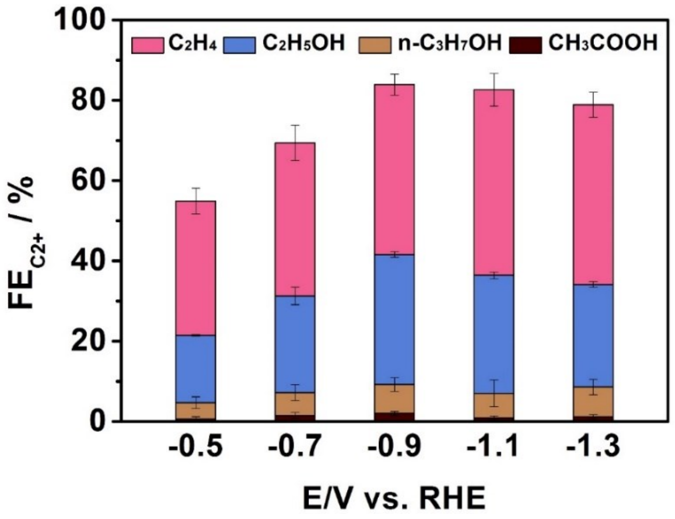

Figure S13. The distribution of C2+ products for Cu-nr-OR.

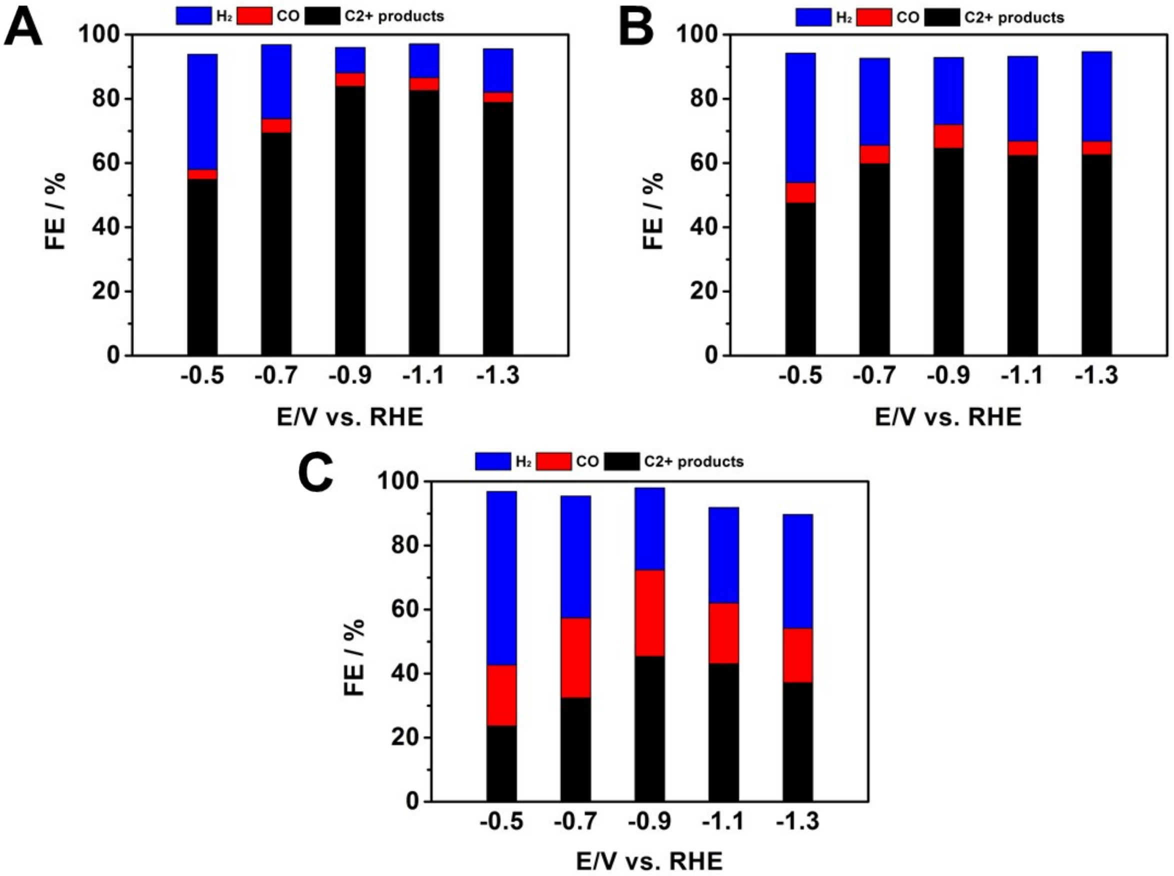

Figure S14. (A) The total FE of Cu-nr-OR. (B) The total FE of Cu-nr. (C) The total FE of Cu-np.

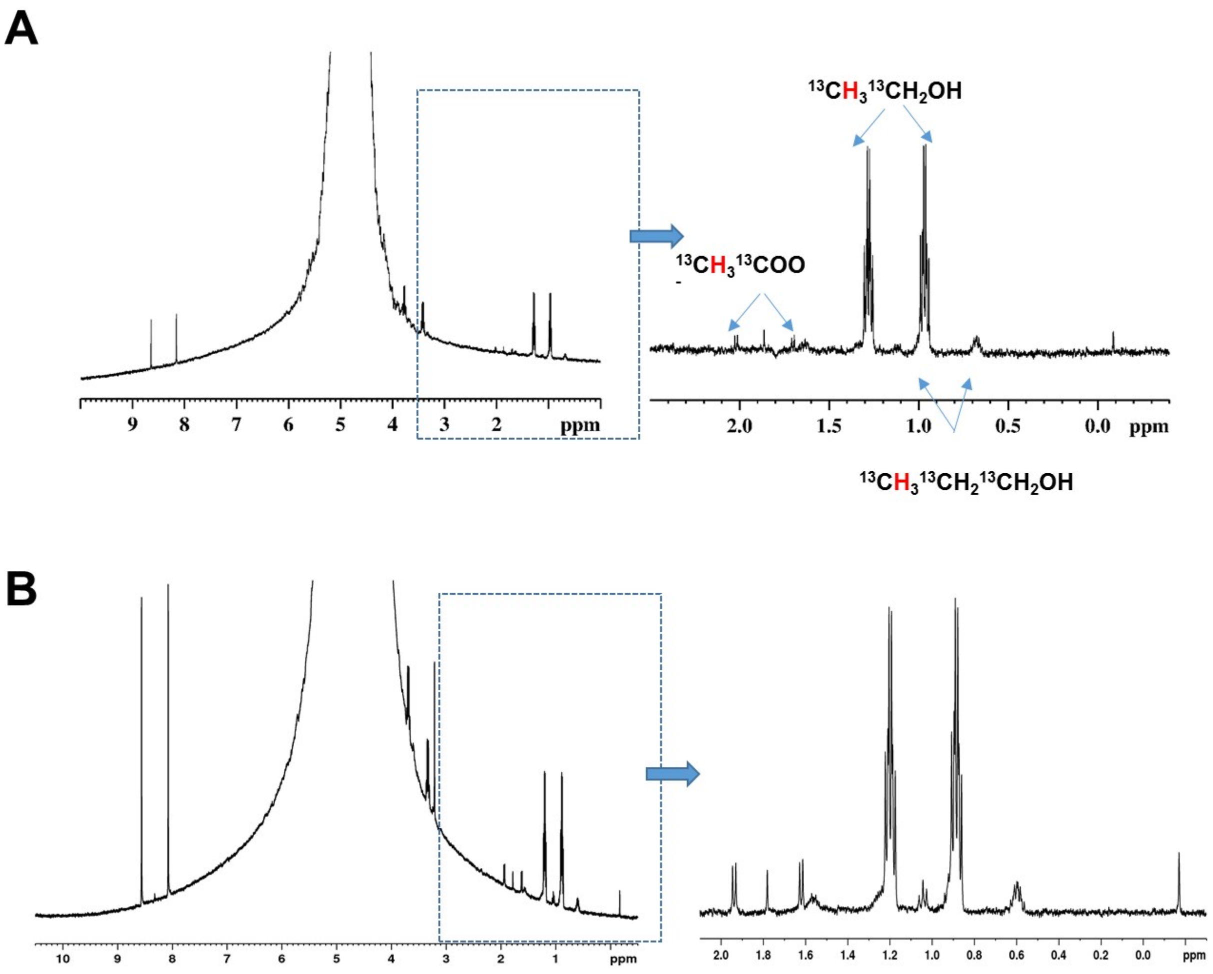

Figure S15. (A) ^{1}H NMR spectra of the liquid products using $^{13}CO_2$ as gas source over Cu-nr-OR for 30 min. (B) ^{1}H NMR spectra of the liquid products using $^{13}CO_2$ as gas source over Cu-nr-OR for 60 min.

We can observe that the H signal of the products spilts into two group peaks, and the singal of n-propanol were located at 0.6ppm and 1.05ppm, when using $^{13}CO_2$ as reactant. Due to the signal of impurity of DSS was also located at 0.6 ppm, which was overlapped with the signal of n-propanol, thus the internal standard cannot be used in Figure S15. In addition, we can observe that the intensity of products increased with the reaction time..

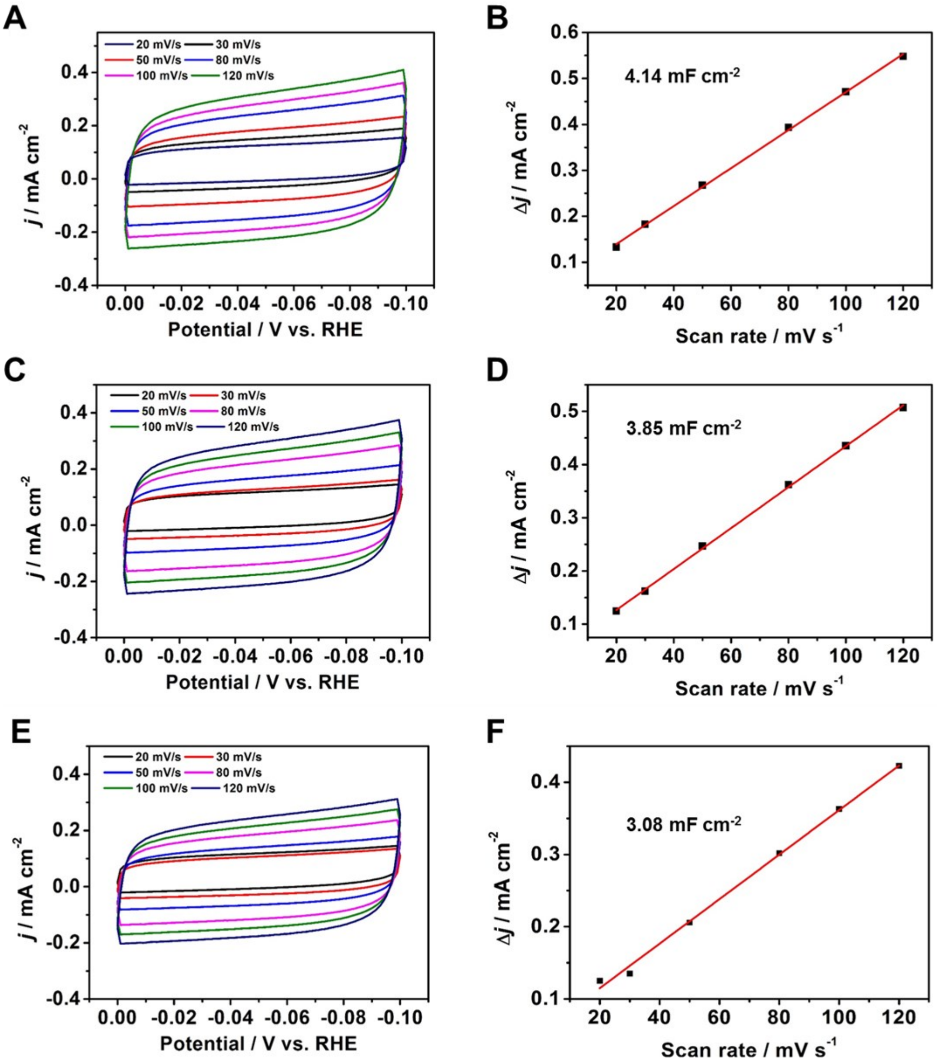

Figure S16. (A) The cyclic voltammetry at different scan rates over Cu-nr; (B) The charging current density differences plotted against the scan rates over Cu-nr; (C) The cyclic voltammetry at different scan rates over Cu-nr-OR; (D) The charging current density differences plotted against the scan rates of Cu-nr-OR; (E) The cyclic voltammetry at different scan rates over Cu-np; (F) The charging current density differences plotted against the scan rates of Cu-np.

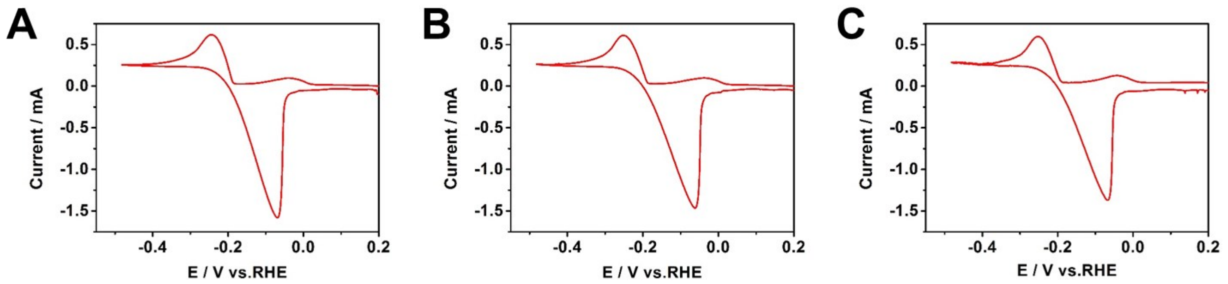

Figure S17. (A) The cyclic voltammetry over Cu-nr-OR in 100 mM $HClO_4$ + 1 mM $Pb(ClO_4)_2$. (B) The cyclic voltammetry over Cu-nr in 100 mM $HClO_4$ + 1 mM $Pb(ClO_4)_2$. (C) The cyclic voltammetry over Cu-np in 100 mM $HClO_4$ + 1 mM $Pb(ClO_4)_2$.

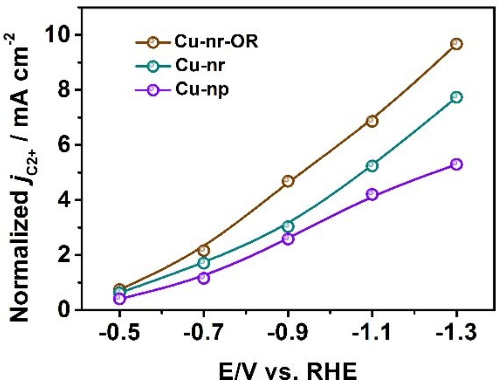

Figure S18. The normalized partial current density for C2+ products over different catalysts by ECSA in 1 M KOH solution.

\

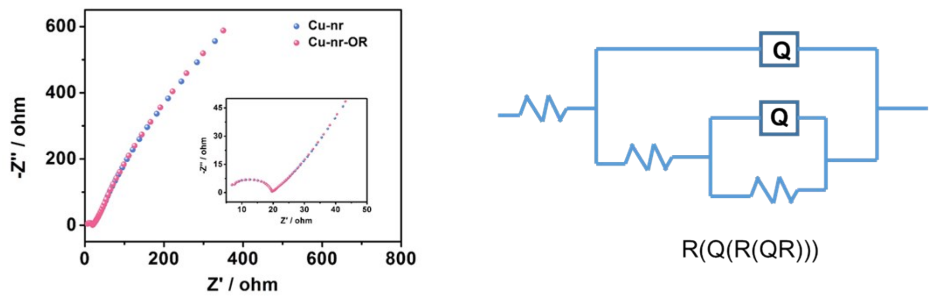

Figure S19. Nyquist plots for different electrodes in CO_2-saturated 1 M KOH electrolyte.

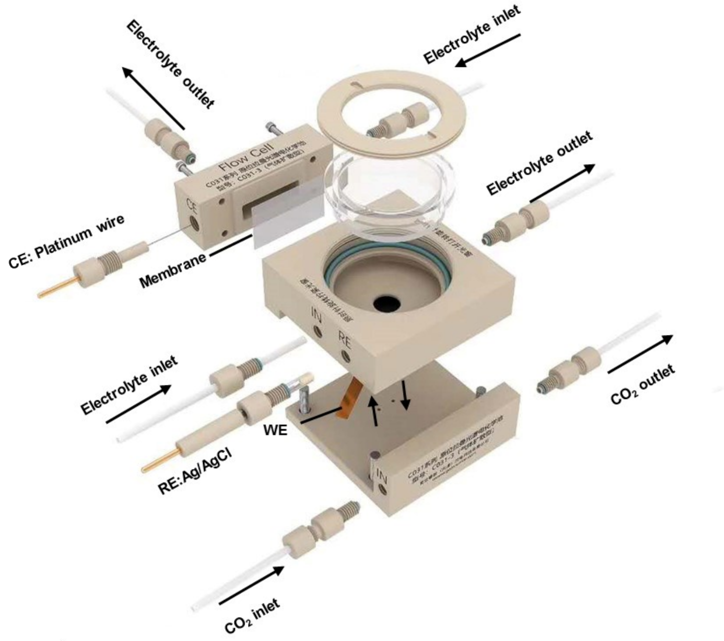

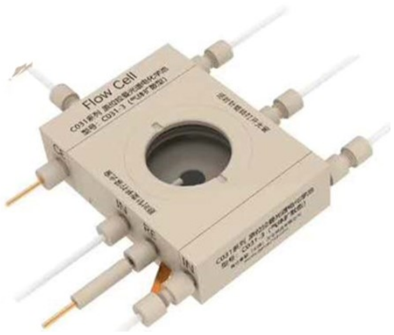

Figure S20. In situ electrochemical spectral cell for SERS test.

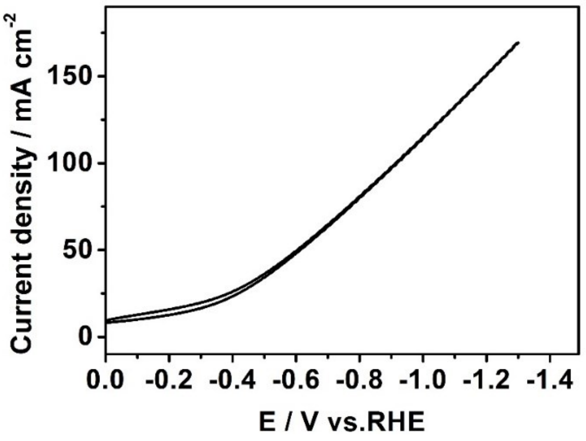

Figure S21. The cyclic voltammetry over Cu-nr-OR in 1MKOH

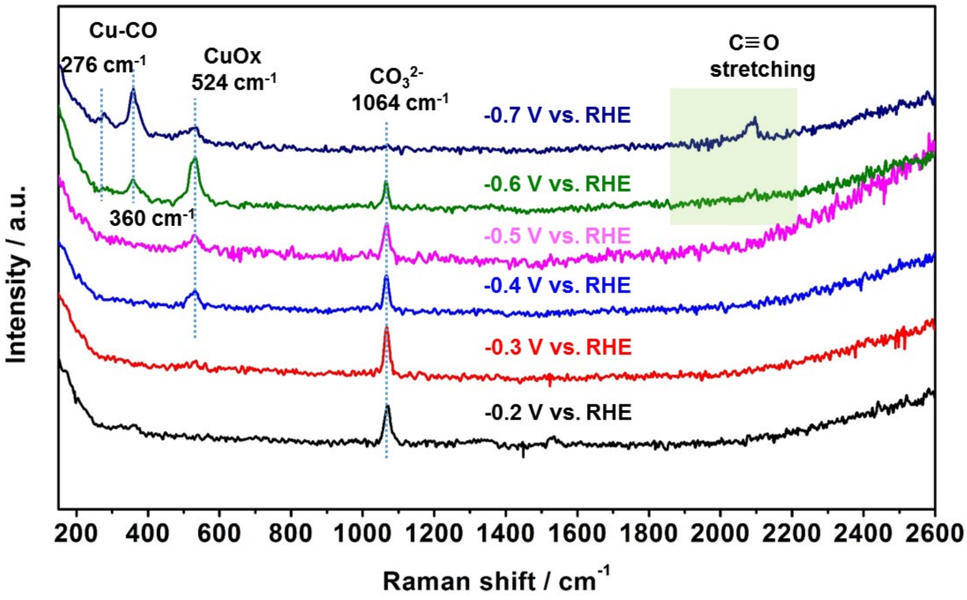

Figure S22. The in-situ SERS for Cu-nr with D_2O as electrolyte at different potentials during CO_2RR.

For the SERS measurement in D_2O, the KOH dissolved in D_2O as an electrolyte.

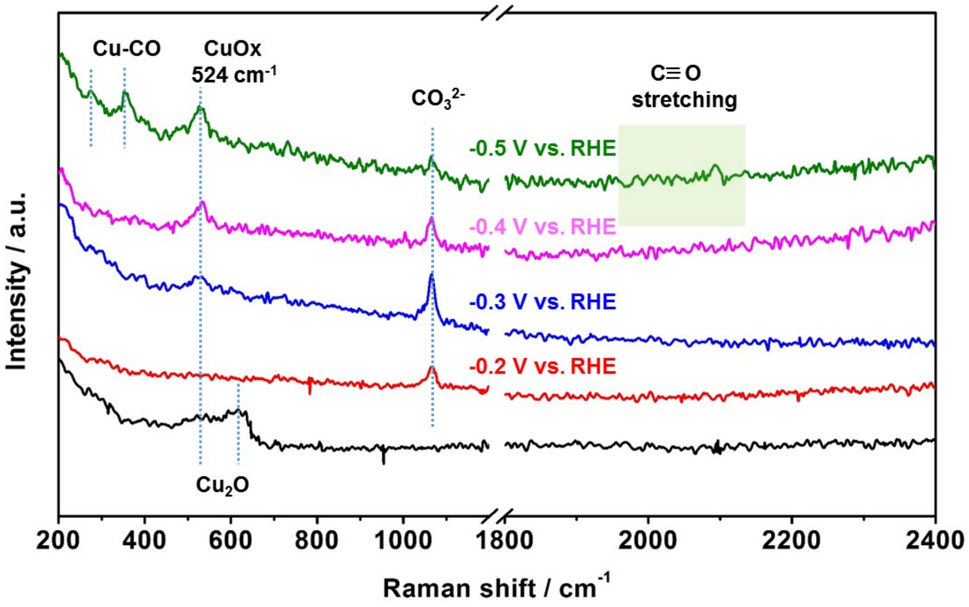

Figure S23. The in-situ SERS for Cu-nr-OR with $H_2{}^{18}O$ as electrolyte at different potentials during CO_2RR.

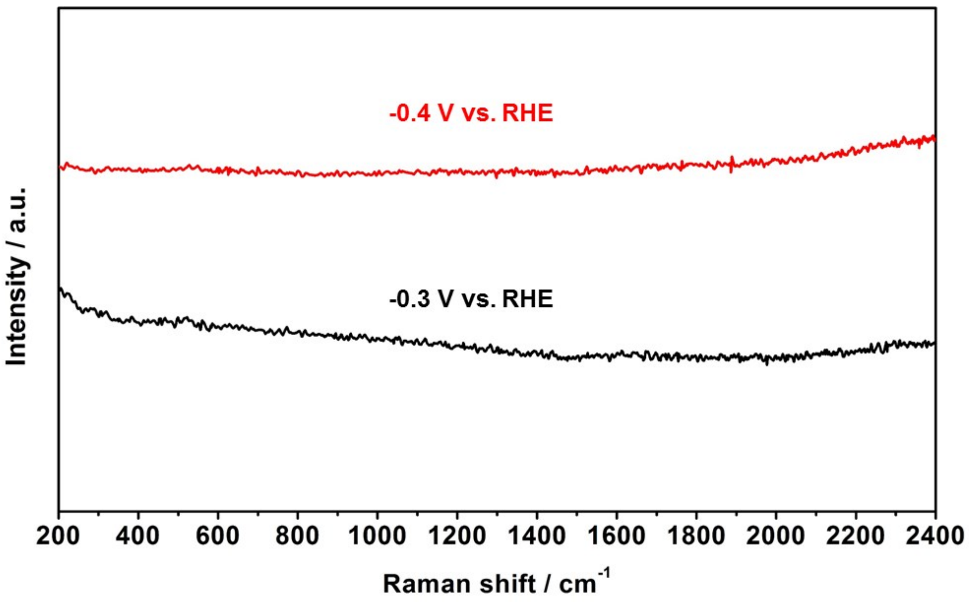

Figure S24. The in-situ SERS for Cu-nr-OR at different potentials in N_2 atmosphere.

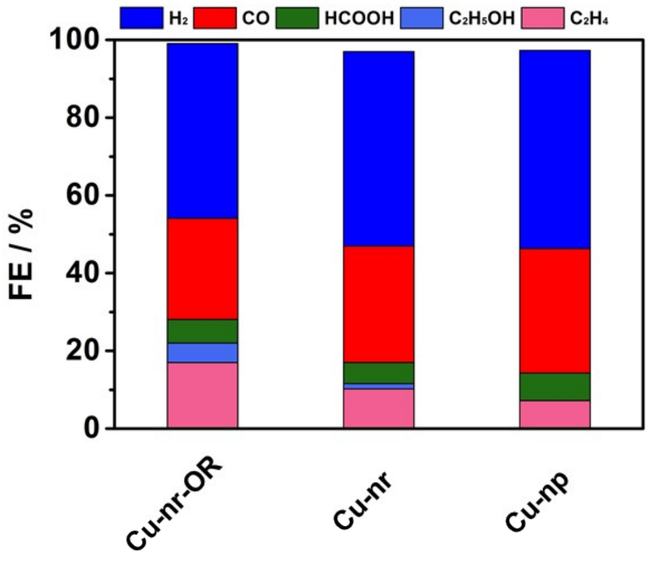

Figure S25. The distribution of products for Cu-nr-OR, Cu-nr and Cu-np at -0.4 V vs. RHE..

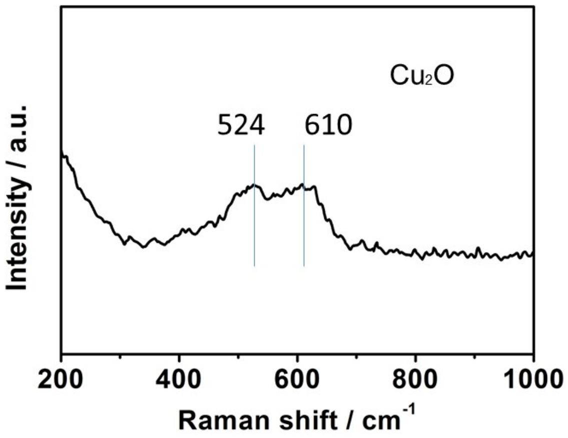

Figure S26. The in-situ SERS for Cu-nr-OR with H_2O as electrolyte after the potential was removed.

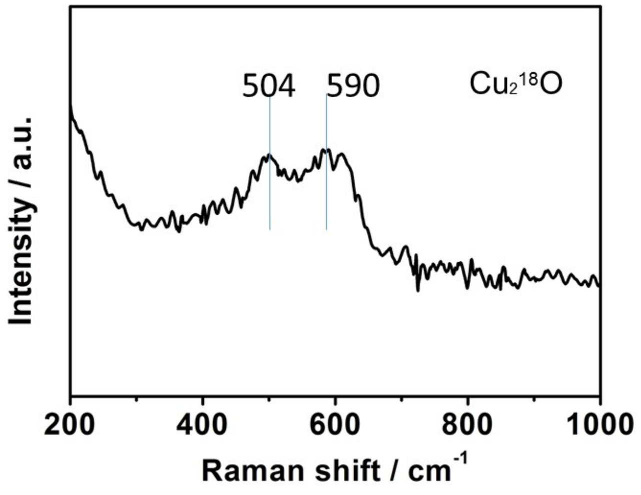

Figure S27. The in-situ SERS for Cu-nr-OR with $H_2{}^{18}O$ as electrolyte after the potential was removed.

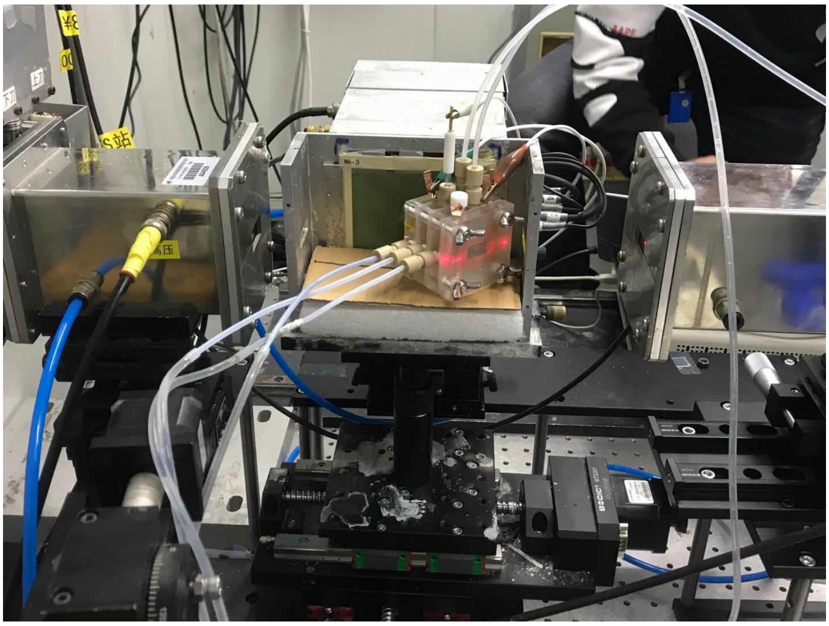

Figure S28. Operando electrochemical cell for XAS test.

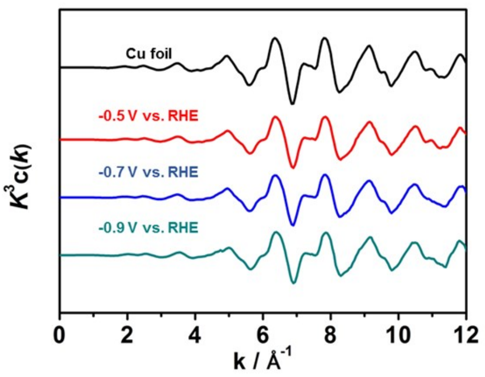

Figure S29. Cu K-edge extended XAFS oscillation function $k^3w(k)$ for Cu-nr-OR at different potentials

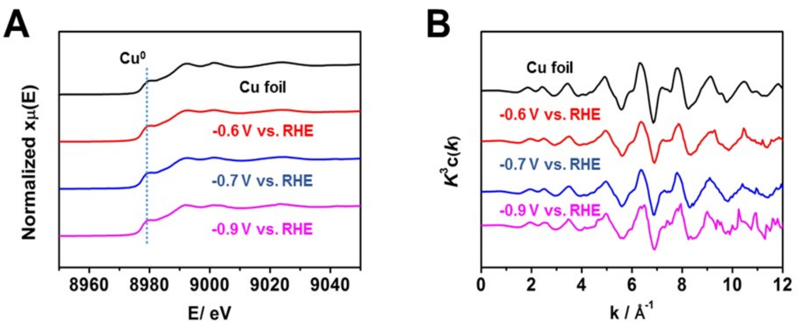

Figure S30. (A) XANES spectra at the Cu K-edge for Cu-nr at different potentials. (B) Cu K-edge extended XAFS oscillation function $k^3w(k)$ for Cu-nr at different potentials. (C) The corresponding Fourier transforms $FT(k^3w(k))$ for Cu-nr at different potentials.

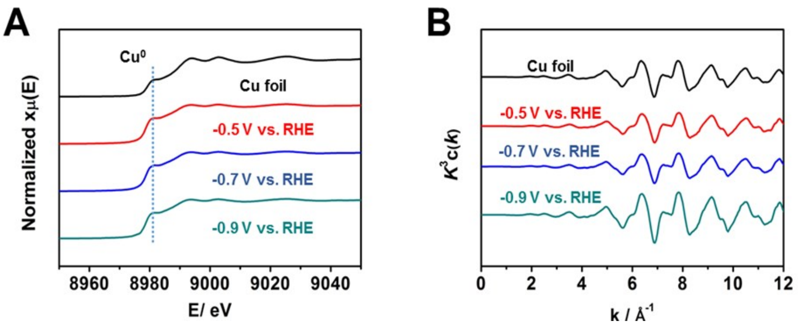

Figure S31. (A) XANES spectra at the Cu K-edge for Cu-np at different potentials. (B) Cu K-edge extended XAFS oscillation function $k^3w(k)$ for Cu-np at different potentials. (C) The corresponding Fourier transforms $FT(k^3w(k))$ for Cu-np at different potentials.

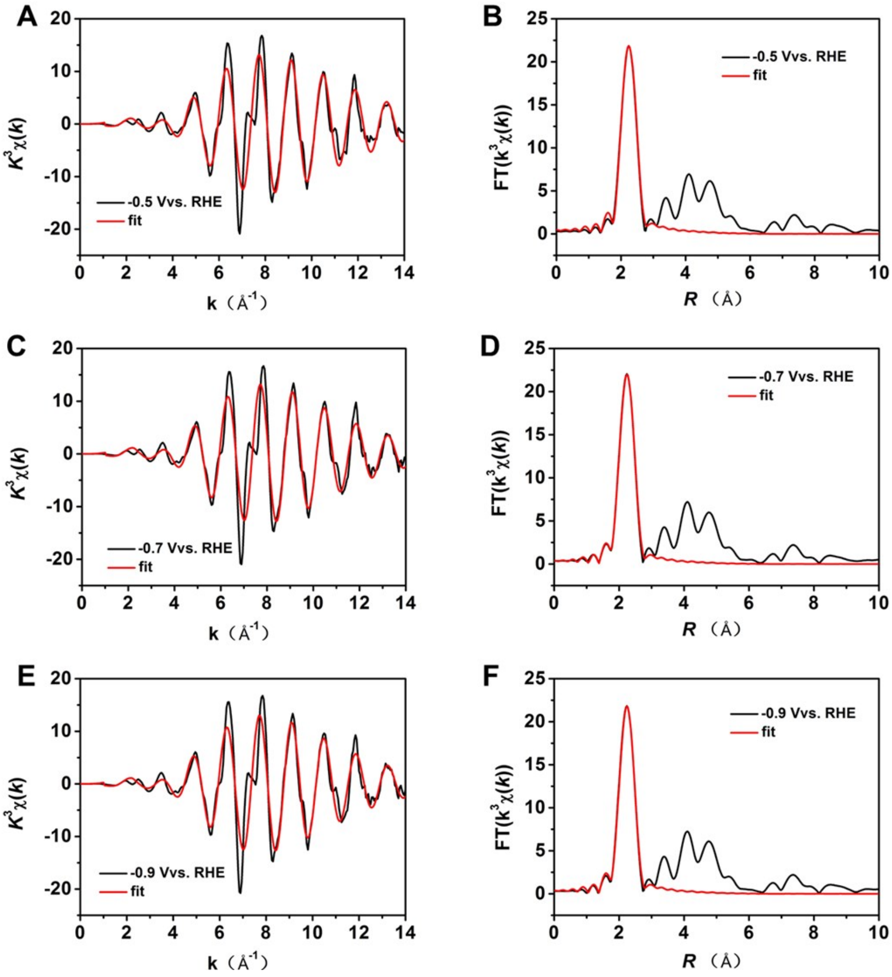

Figure S32. The EXAFS data fitting results of Cu-np at different potentials.

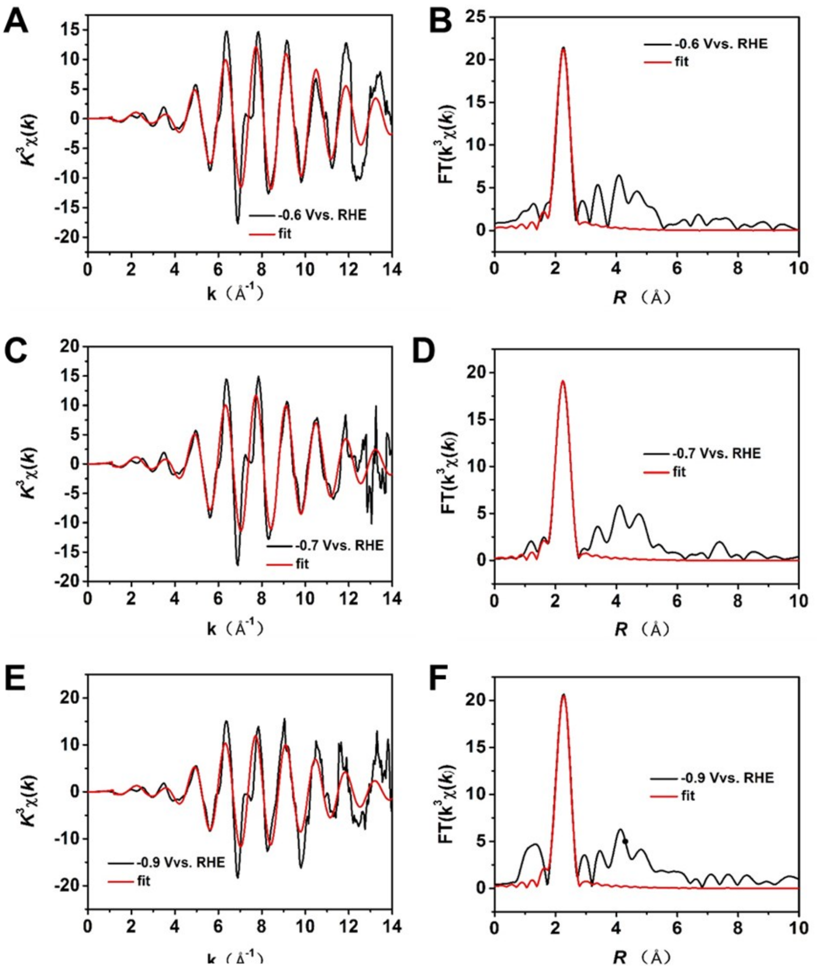

Figure S33. The EXAFS data fitting results of Cu-nr at different potentials.

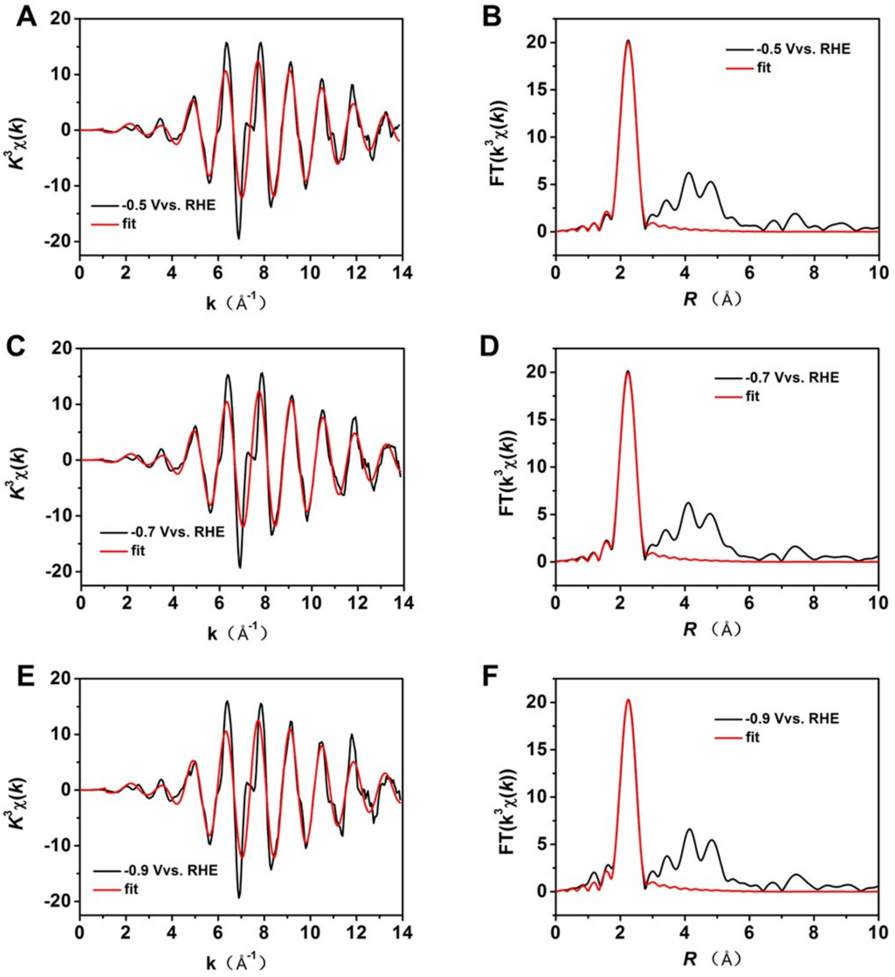

Figure S34. The EXAFS data fitting results of Cu-nr-OR at different potentials.

Supplementary Tables

Table S1. Comparision of C2+ products in CO_2RR on various Cu-based catalysts.

Samples	E vs. RHE	FE_{C2+} (%)	J (mA cm^{-2})	references
Cu-nr-OR	-0.9	83.8	341.5	This work
Cu-nr	-0.9	64.6	271.2	This work
Ce(OH)x/Cu	-0.7	80.3	297	S5
FeTPP[Cl]/Cu	-0.82	85	302.4	S6
Cu-CO_2	-0.71	90	577	S7
CuAg wire	-0.7	85	300	S8
NGQ/Cu-nr	-0.9	80.4	282.1	S2
Cu-chloride	-0.68	84	400	S9
Nanoporous Cu	-0.67	62	653	S1

Table S2. Cu_{ECSA} determined by Pb_{UPD} method for different samples.

Sample	Cu_{ECSA} (cm^2)
Cu-nr-OR	61.2
Cu-nr	57.8
Cu-np	49.2

Table S3. Structural parameters of Cu-nr and Cu-nr-OR at different potentials extracted from the EXAFS fitting.

Sample	Potential V vs.RHE	Scattering pair	CN	R(Å)	$\sigma^2(10^{-3}Å^2)$	ΔE_0(eV)
Cu-nr	-0.6	Cu-Cu	9.8.0±0.8	2.55±0.02	5.4±0.8	3.4±0.3
Cu-nr	-0.7	Cu-Cu	9.8±0.9	2.55±0.02	5.8±0.6	3.4±0.8
Cu-nr	-0.9	Cu-Cu	9.8±0.7	2.55±0.02	6.2±0.7	3.4±0.7
Cu-nr-OR	-0.5	Cu-Cu	9.5±0.6	2.56±0.02	5.1±0.6	3.2±0.6
Cu-nr-OR	-0.7	Cu-Cu	9.5±0.8	2.56±0.02	5.1±0.6	3.2±0.8
Cu-nr-OR	-0.9	Cu-Cu	9.5±0.7	2.56±0.02	5.1±0.7	3.2±0.7
Cu-np	-0.5	Cu-Cu	9.8±0.7	2.56±0.02	5.4±0.6	3.2±0.7
Cu-np	-0.7	Cu-Cu	9.9±0.8	2.56±0.02	5.8±0.7	3.2±0.8
Cu-np	-0.9	Cu-Cu	9.8±0.7	2.56±0.02	6.1±0.6	3.2±0.7

S_0^2 is the amplitude reduction factor S_0^2=0.85; CN is the coordination number; R is interatomic distance (the bond length between central atoms and surrounding coordination atoms); σ^2 is Debye-Waller factor (a measure of thermal and static disorder in absorber-scatterer distances); ΔE_0 is edge-energy shift (the difference between the zero kinetic energy value of the sample and that of the theoretical model). R factor is used to value the goodness of the fitting.

References

S1 J. J. Lv, M. Jouny, W. Luc, W. Zhu, J. J. Zhu, F. Jiao, *Adv. Mater.* **2018**, *30*, e1803111.

S2 C. Chen, X. Yan, S. Liu, Y. Wu, Q. Wan, X. Sun, Q. Zhu, H. Liu, J. Ma, L. Zheng, H. Wu, B. Han, *Angew. Chem. Int. Ed.* **2020***, 59, 16459-16464..*

S3 G. Kresse, J. Furthmüller, *Phys. Rev. B,* **1996**, *54*, 11169-11186.

S4 G. Kresse, J. Hafner, *Ab initio. Phys. Rev. B,* **1994***,* 49, 14251-14269.

S5 M. Luo, Z. Wang, Y. C. Li, J. Li, F. Li, Y. Lum, D. H. Nam, B. Chen, J. Wicks, A. Xu, T. Zhuang, W. R. Leow, X. Wang, C. T. Dinh, Y. Wang, Y. Wang, D. Sinton, E. H. Sargent, *Nat. Commun.* **2019**, *10*, 5814.

S6 F. Li, Y. C. Li, Z. Wang, J. Li, D.-H. Nam, Y. Lum, M. Luo, X. Wang, A. Ozden, S.-F. Hung, B. Chen, Y. Wang, J. Wicks, Y. Xu, Y. Li, C. M. Gabardo, C.-T. Dinh, Y. Wang, T.-T. Zhuang, D. Sinton, E. H. Sargent, *Nat. Catal.* **2020**, *3*, 75-82.

S7 Y. Wang, Z. Wang, C.-T. Dinh, J. Li, A. Ozden, M. Golam Kibria, A. Seifitokaldani, C.-S. Tan, C. M. Gabardo, M. Luo, H. Zhou, F. Li, Y. Lum, C. McCallum, Y. Xu, M. Liu, A. Proppe, A. Johnston, P. Todorovic, T.-T. Zhuang, D. Sinton, S. O. Kelley, E. H. Sargent, *Nat. Catal.* **2020**, *3*, 98-106.

S8 T. T. H. Hoang, S. Verma, S. Ma, T. T. Fister, J. Timoshenko, A. I. Frenkel, P. J. A. Kenis, A. A. Gewirth, *J. Am. Chem. Soc.* **2018**, *140*, 5791-5797.

S9 M. G. Kibria, C. T. Dinh, A. Seifitokaldani, P. De Luna, T. Burdyny, R. Quintero-Bermudez, M. B. Ross, O. S. Bushuyev, F. P. Garcia de Arquer, P. Yang, D. Sinton, E. H. Sargent, *Adv Mater* **2018**, e1804867.
